# Beyond Precipitation: Physiographic Gradients Dictate the Relative Importance of Environmental Drivers on Savanna Vegetation

**DOI:** 10.1371/journal.pone.0072348

**Published:** 2013-08-30

**Authors:** Miguel A. Campo-Bescós, Rafael Muñoz-Carpena, David A. Kaplan, Jane Southworth, Likai Zhu, Peter R. Waylen

**Affiliations:** 1 Agricultural and Biological Engineering Department, University of Florida, Gainesville, Florida, United States of America; 2 Environmental Engineering Sciences Department, University of Florida, Gainesville, Florida, United States of America; 3 Geography Department, University of Florida, Gainesville, Florida, United States of America; 4 Projects and Rural Engineering Department, Public University of Navarre, Ed. Los Olivos, Pamplona, Spain; Lakehead University, Canada

## Abstract

**Background:**

Understanding the drivers of large-scale vegetation change is critical to managing landscapes and key to predicting how projected climate and land use changes will affect regional vegetation patterns. This study aimed to improve our understanding of the role, magnitude and spatial distribution of the key environmental factors driving vegetation change in southern African savanna, and how they vary across physiographic gradients.

**Methodology/Principal Findings:**

We applied Dynamic Factor Analysis (DFA), a multivariate times series dimension reduction technique to ten years of monthly remote sensing data (MODIS-derived normalized difference vegetation index, NDVI) and a suite of environmental covariates: precipitation, mean and maximum temperature, soil moisture, relative humidity, fire and potential evapotranspiration. Monthly NDVI was described by cyclic seasonal variation with distinct spatiotemporal patterns in different physiographic regions. Results support existing work emphasizing the importance of precipitation, soil moisture and fire on NDVI, but also reveal overlooked effects of temperature and evapotranspiration, particularly in regions with higher mean annual precipitation. Critically, spatial distributions of the weights of environmental covariates point to a transition in the importance of precipitation and soil moisture (strongest in grass-dominated regions with precipitation<750 mm) to fire, potential evapotranspiration, and temperature (strongest in tree-dominated regions with precipitation>950 mm).

**Conclusions/Significance:**

We quantified the combined spatiotemporal effects of an available suite of environmental drivers on NDVI across a large and diverse savanna region. The analysis supports known drivers of savanna vegetation but also uncovers important roles of temperature and evapotranspiration. Results highlight the utility of applying the DFA approach to remote sensing products for regional analyses of landscape change in the context of global environmental change. With the dramatic increase in global change research, this methodology augurs well for further development and application of spatially explicit time series modeling to studies at the intersection of ecology and remote sensing.

## Introduction

Understanding the drivers of large-scale vegetation change is critical to managing landscapes for the mutual benefit of human and natural systems. Tropical and sub-tropical southern Africa is dominated by the presence of savannas characterized by the coexistence of trees and grasses. Savanna is a relatively productive ecosystem; the entire African savanna biome accounts for 13.6% of global Net Primary Production NPP [1]. Long-term changes in ecosystem structure and productivity is thought to be driven by a combination of biotic (including human) and abiotic drivers [2]–[7], and may represent irreversible landscape degradation [8]. Degradation of southern African savanna [9] is usually represented on the landscape as a shift from grass- and tree-dominated landscapes to less biologically productive ones dominated by scrub [10]–[12]. Given this trend, understanding the spatial and temporal dynamics of vegetation change and identifying the main drivers of vegetation transition are critically important for land management, particularly in light of significant climate variability and possible directional climate change expected in the region [13], [14].

The critical importance of single factors like water [6], [15]–[17], specifically mean annual precipitation (MAP) [3], [4], [7], is recognized for savanna vegetation, although its role differs across vegetation types and biomes, and it’s effects are typically analyzed at annual or longer time steps. Rainfall stimulates green-up onset and determines the duration of growth and flowering of some plants, and its distribution is critical to vegetation germination, growth, and biomass [18]. Other individual factors like soils, nutrients, herbivory, and land use (mainly grazing activities) contribute to local patterns of savanna ecosystem structure at longer time frames [4], [19]. However, a simultaneous spatial and temporal analysis of the dynamics of the coupled relationship among environmental covariates controlling savanna vegetation has not been explored, specially at a finer temporal resolution (i.e. monthly). Integrated analysis of environmental factors is critically important for explaining abiotic-biotic interactions over large spatial scales, where the relative importance of abiotic drivers on vegetation may vary between different physiographic regions. Thus, a method for simultaneously identifying spatial patterns of the importance of multiple, dynamic explanatory variables–while accounting for unexplained, but shared, temporally varying trends–is required to improve understanding of the factors controlling vegetation response.

Remote sensing has provided a powerful instrument to observe, monitor and characterize landscape changes, since it is able to offer repeated measurements of large terrestrial areas at long temporal scales [14], [20]–[23]. Satellite-derived vegetation indices such as the normalized difference vegetation index (NDVI) and the enhanced vegetation index (EVI) have been closely linked with fraction of green vegetation, leaf area index, and vegetation primary production [24]. Time series approaches to analyzing continuous remote sensing variables are being adopted to advance understanding of intra- and inter-annual variations in vegetation and to derive and examine the relationships between vegetation growth and drivers of change [3], [4], [6], [25]–[34]. Application of wavelet analysis [35] and Fourier analysis [36]–[38] show promise in elucidating patterns of land cover variation and individual drivers of change, but rarely are multiple, spatiotemporally variable drivers of landscape change assessed simultaneously.

To address this challenge, we applied Dynamic Factor Analysis (DFA), a multivariate times series dimension reduction technique, to investigate vegetation dynamics (via NDVI) across three large watersheds in southern Africa. DFA models temporal variation in observed data series (response variable) as linear combinations of one or more common trends (representing *unexplained variability*) and zero or more explanatory variables (representing *explained variability*). DFA was initially developed to analyze economic time series [39]–[42] and was later extended to include explanatory variables in ecological predictions, like modeling the dynamics of squid [43] and commercial fisheries [44]–[49]. DFA has been successfully applied to improve understanding of variation in groundwater levels [50]–[52], soil moisture dynamics [53], [54], interactions between hydrological variables and groundwater quality trends [55], [56], annual maximum precipitation [57], and air quality [58]. Recently, DFA has been used to link radial tree growth and climate [59], [60].

**Figure 1:**
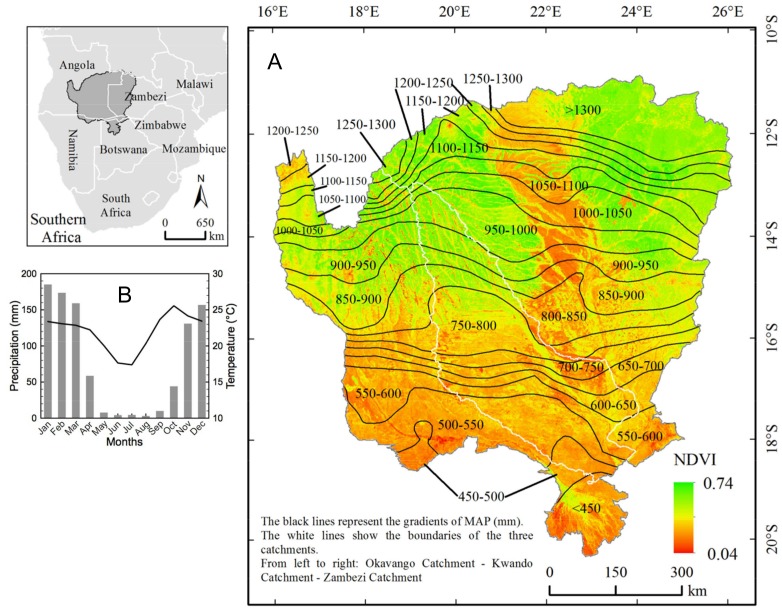
Geography of the study area with (A) mean annual NDVI and precipitation and (B) climograph. Spatial pattern of mean annual NDVI across the study area, derived from monthly MODIS NDVI data from 2001 to 2010. The subset polygons correspond to mean annual precipitation intervals. The inset map shows the geographic location of the study area in southern Africa.

In this study, we applied DFA to the temporal and spatial interactions between monthly NDVI and a suite of monthly environmental covariates across the Okavango, Kwando, and upper Zambezi watersheds, representing a dynamic savanna landscape in southern Africa (Fig. 1**A**). The specific objective of this study was to identify the most important physical drivers of vegetation cover in the region and how they vary across physiographic gradients at a monthly temporal scale. This analysis supports the role of known drivers of savanna vegetation but also uncovered the important roles of temperature and evapotranspiration at this finer temporal resolution. Understanding the shifts in importance of combined drivers of large-scale vegetation change is critical to managing landscapes in the context of projected climate and land use changes.

## Results

### Experimental Time Series

**Figure 2:**
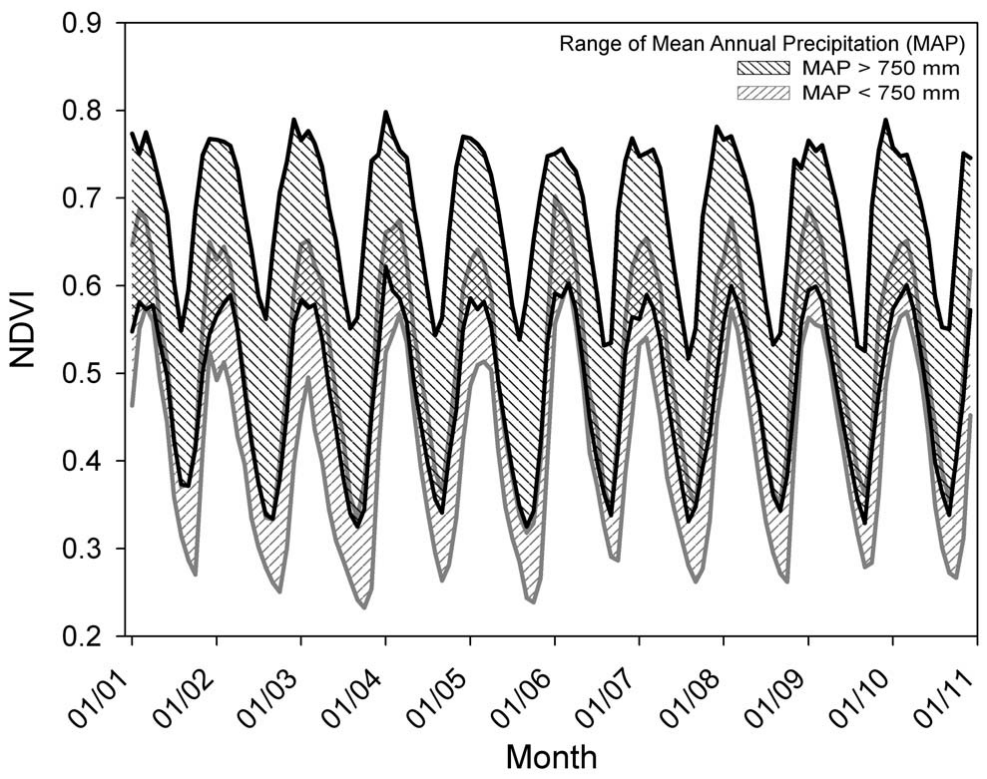
Variability of observed NDVI for the study region for two mean annual precipitation (MAP) ranges.

Monthly NDVI data collected between 2001 and 2010 showed relatively consistent seasonal cycling, typical for this region [61], across all data analysis precipitation polygons depicted in Fig. 1**A**. However, variations in the magnitude of NDVI indicate considerable spatial variation in vegetation, with highest values in the more humid north (MAP>750 mm) (Fig. 2). Moreover, while rising and declining phases of NDVI are generally parallel across different precipitation polygons, vegetation dynamics differ spatially and temporally during periods of NDVI minima and maxima (Fig. 2). For example, the timing of summer NDVI peaks varies across physiographic regions receiving different amounts of MAP. Additionally, years with lower summertime NDVI maxima (e.g. 2003 and 2005) in regions with low MAP do not necessarily correspond to similarly low maxima in more humid regions.

### Candidate Explanatory Variables

The application of the collinearity test (VIF<10, Methods section) resulted in the selection of six CEVs for further investigation (see details in Appendix S1, Table A2): precipitation (P), mean temperature (T), maximum temperature (M), soil moisture (S), fire (F) and potential evapotranspiration (E) (Table 1). Each CEV consisted of monthly time series from each of the 48 analysis polygons, yielding a total of 288 CEVs; this set of explanatory time series was reduced by performing an initial DFA on each CEV. In all cases, the inclusion of the area-weighted average time series for the whole study region as an explanatory variable yielded satisfactory reduced models, and resulted in minimum BIC when combined with a single common trend representing local “anomalies” from the average time series (Table S1). Sufficiency of these reduced CEVs were supported by good model fitting (global *C_eff_*>0.90 for all response variables except fire) and a marginal increase of *C_eff_* for DFMs with higher numbers of trends (Table S1). This indicates that the majority of observed variance in each set of 48 spatially distinct CEVs was well accounted by the global area-weighted average and a single trend (anomaly).

**Table 1:** Data sets used in DFA analysis and their sources.

Data set	Symbol	Source
Monthly MODIS NDVI (MOD13A3)	NDVI	http://reverb.echo.nasa.gov
Monthly MODIS Thermal Anomalies & Fire (MOD14A2)	F	http://reverb.echo.nasa.gov
Matsuura and Willmott’ s Monthly Precipitation	P	http://climate.geog.udel.edu/~climate/html_pages/download.html#P2011
Matsuura and Willmott’ s Monthly Mean andMaximum Temperature	T, M	http://climate.geog.udel.edu/~climate/html_pages/download.html#P2011
CPC Monthly Soil Moisture	S	http://www.esrl.noaa.gov/psd/data/gridded/tables/monthly.html
NCEP-DOE Reanalysis II MonthlyPotential Evapotranspiration	E	http://www.esrl.noaa.gov/psd/data/gridded/data.ncep.reanalysis2.gaussian.html

**Figure 3:**
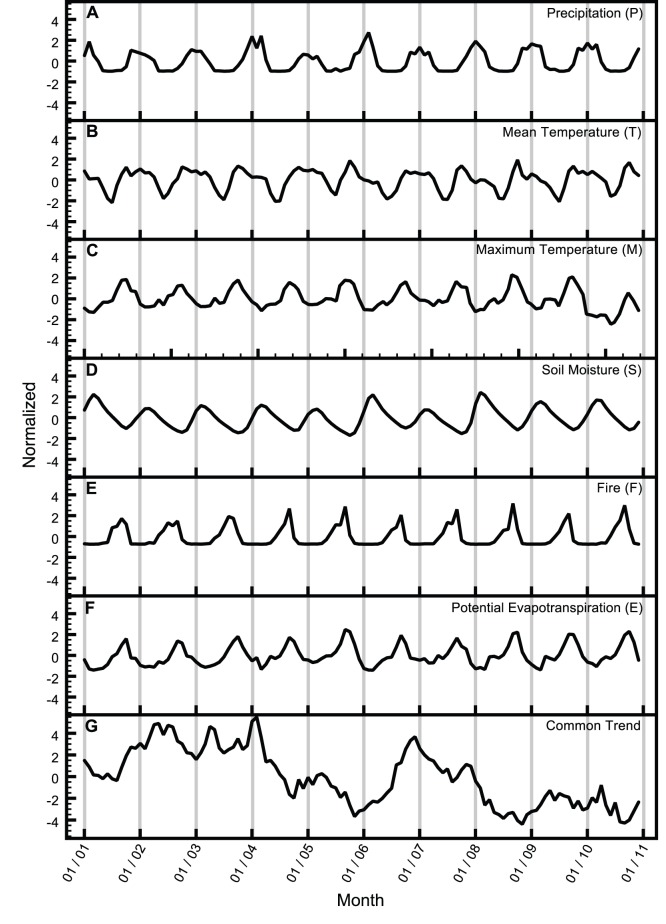
Normalized, area-weighted average explanatory variables used in the study. (**A**) precipitation, P; (**B**) mean temperature, T; (**C**) maximum temperature, M; (**D**) soil moisture, S; (**E**) fire, F; and (**F**) potential evapotranspiration, E. The common trend (**G**) for model II (FSPT) is shown for comparison.

Normalized, area-weighted average time series of the CEVs are presented in Fig. 3**A**–**F**. While all CEVs show an annual cycle, each is unique in its timing of seasonal maxima/minima and rates of rise and decline. For example, precipitation, mean temperature, and soil moisture are generally in phase (i.e. with winter maxima), however correlation between the timing of precipitation and mean temperature maxima is inconsistent across years, and the rate of declining soil moisture is attenuated and delayed with respect to precipitation. Similarly, while fire, maximum temperature and E share the general timing of summer maxima, the E signal is better resolved in winter months, while the fire signal disappears (i.e. time when the burned area is essentially zero). Due to these distinct patterns of temporal variation, no combination of the six explanatory variables exceeded the collinearity threshold (VIF<10) and the set was deemed suitable for use as CEVs in the exploration of NDVI models.

### DFA with no Explanatory Variables (Model I)

As a baseline for comparison, DFA was first applied to model the 48 NDVI time series (response variable) using only an increasing number of common trends (Model I). BIC was minimized with one trend (*M* = 1), and addition of extra trends impact on model performance minimally (Table 2). Overall C*_eff_* was 0.90 for Model I, ranging between 0.78 and 0.96 across precipitation polygons (Table 2). The single common trend in Model I illustrates shared variability across the 48 input time series and may be viewed as a general signature of NDVI across the domain, integrating environmental factors that influence vegetation dynamics. High positive correlations (0.88≤*ρ_1,n≤_*0.98) between the common trend and the 48 NDVI response time series indicates that the model captures the principal variability of NDVI across the region. However, since this trend represents a latent effect (i.e. unexplained variance), it does not reveal which biophysical variables are actually driving NDVI variations.

**Table 2:** Selected results of the dynamic factor analysis of Normalized Difference Vegetation Index (NDVI).

Model^a^	No. oftrends (M)	Explanatory variables	No. of parameters	BIC^b^	C_eff_ c	*p*-value^d^	*ρ_1,n_* e
I	**1**	**−**	1272	−**1854**	**0.90 (0.78–0.96)**	+	0.95 (0.88–0.98)
	2	**−**	1319	−1668	0.91 (0.79–0.96)	+	
	3	**−**	1365	−1491	0.91 (0.79–0.96)	+	
II	1	P	1320	−1683	0.86 (0.70–0.94)	+	0.73 (0.53–0.85)
	2	P	1367	−1504	0.87 (0.72–0.94)	+	
	3	P	1413	−1308	0.87 (0.72–0.93)	+	
	1	T	1320	−1712	0.91 (0.79–0.96)	+	0.94 (0.84–0.98)
	2	T	1367	−1566	0.91 (0.79–0.95)	+	
	3	T	1413	−1373	0.91 (0.79–0.96)	+	
	1	M	1320	−1704	0.91 (0.79–0.96)	+	0.86 (0.77–0.92)
	2	M	1367	−1521	0.90 (0.78–0.94)	+	
	3	M	1413	−1316	0.90 (0.80–0.94)	+	
	1	S	1320	−1807	0.89 (0.79–0.93)	+	0.74 (0.55–0.88)
	2	S	1367	−1626	0.89 (0.79–0.93)	+	
	3	S	1413	−1404	0.91 (0.80–0.95)	+	
	1	F	1320	−1706	0.87 (0.74–0.92)	+	0.77 (0.60–0.90)
	2	F	1367	−1519	0.88 (0.78–0.93)	+	
	3	F	1413	−1329	0.88 (0.81–0.92)	+	
	1	E	1320	−1758	0.88 (0.73–0.95)	+	0.76 (0.70–0.82)
	2	E	1367	−1592	0.89 (0.78–0.93)	+	
	3	E	1413	−1398	0.92 (0.85–0.96)	+	
	1	F S	1368	−1648	0.91 (0.84–0.94)	+	0.61 (0.45–0.71)
	2	F S	1415	−1465	0.89 (0.83–0.94)	+	
	3	F S	1461	−1304	0.90 (0.81–0.93)	+	
	1	F S P	1416	−1510	0.90 (0.83–0.94)	+	0.26 (0.06–0.35)
	2	F S P	1463	−1312	0.90 (0.83–0.94)	+	
	3	F S P	1509	−1121	0.91 (0.83–0.94)	+	
	**1**	**F S P T**	1464	−**1376**	**0.91 (0.88–0.93)**	**++**	0.09 (−0.09–0.20)
	2	F S P T	1511	−1138	0.92 (0.88–0.94)	++	
	3	F S P T	1557	−937	0.93 (0.88–0.95)	++	
	1	F S P T E	1512	−1048	0.93 (0.88–0.95)	++	0.13 (−0.01–0.27)
	2	F S P T E	1559	−869	0.93 (0.88–0.95)	++	
	3	F S P T E	1605	−682	0.93 (0.88–0.95)	++	
III	0	F	96	−6094	0.70 (0.51–0.81)	**−**	
	0	F S	144	−8719	0.82 (0.70–0.87)	+	
	0	F S P	192	−10975	0.89 (0.81–0.92)	+	
	0	F S P T	240	−11391	0.90 (0.86–0.92)	++	
	**0**	**F S P T E**	288	−**11719**	**0.91 (0.88–0.93)**	**++**	

a Model I: only common trends (unexplained variability), Model II: with common trends and explanatory variables (explained variability), and Model III: only explanatory variables (from multiple regression);

b BIC: Bayesian Information Criterion;

c C_eff_: Nash-Sutcliffe coefficient of efficiency. Values presented are calculated for the whole area (values in parenthesis represent range for polygons);

d Classification of goodness-of-fitness results based on statistical significance (*p* = 0.01) for the polygon with the lowest fit in the region: −Unsatisfactory, +acceptable, ++good [85];

e Canonical correlation coefficients for the first common trend of Models I and II.

### DFA with Explanatory Variables (Model II)

Next, NDVI was modeled as combinations of common trends and selected CEVs to reduce model reliance on the unexplained variability identified in Model I. Based on the reduction of CEVs, combinations of area-weighted average and “anomaly” CEV time series were evaluated in a factorial manner to determine “best” model fits (273 DFMs explored; Table S2). The best-performing models employed a single trend, and none of the best DFMs included CEV “anomaly” time series (see Table S2), suggesting that the combinations of *average* environmental drivers (Fig. 3**A**–**F**) adequately captured spatio-temporal variation of NDVI.

Systematic application of a multi-criteria model selection process (see Methods section) led to the final selection of Model II with one common trend and 4 explanatory variables (fire, soil moisture, precipitation, and mean temperature) (shown in Fig. 3 and bold text in Table 2). This model minimized BIC amongst all DFMs with *M* = 1 and *K* = 4 (Table S2) and had “good” model performance with an overall C*_eff_* = 0.91. Moreover, model performance for the worst-performing polygon was substantially improved (from C*_eff_* = 0.79 with K = 1 to 0.88 with K = 4). Finally, the majority of the model’s explanatory power shifted from the common trend to the explanatory variables, with average canonical cross-correlation between response and trend (*ρ_1,n_*) reduced from 0.94 to 0.09 for *K* = 1 to 4, respectively.

### Multi-linear Regression (Model III)

**Figure 4:**
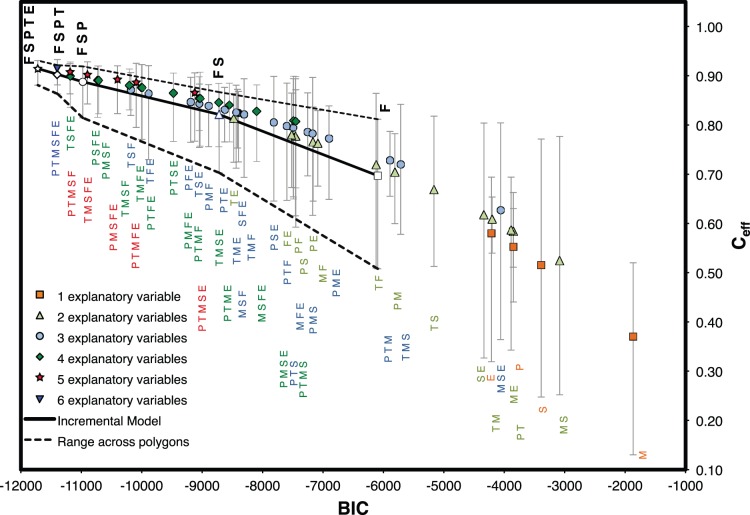
Incremental improvement of model III performance with the addition of the explanatory variables. Variables are precipitation, P; mean temperature, T; maximum temperature, M; soil moisture, S; fire, F; and potential evapotranspiration, E. Best models are shown in bold with white symbols (F, FS, FSP, FSPT, FSPTE), solid lines show weighted averages and dashed lines the range across the spatial domain (C*_eff_*: Nash-Sutcliffe Coefficient; BIC: Bayesian Information Criterion).

**Figure 5:**
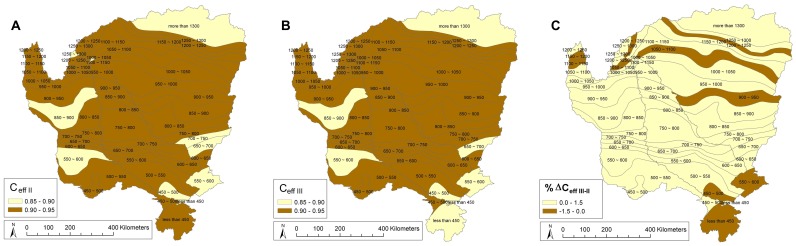
Spatial distribution of goodness-of-fit of the NDVI models. (**A**) final exploratory model II (FSPT) and (**B**) model III (FSPTE) with (**C**) highlighting the changes in goodness-of-fit between model III and II (% ΔC_eff III - II_ = 100*[C_eff III_ - C_eff II_]/C_eff III_), average value = 0.40, max = 1.40 and min = −0.70. (C*_eff_*: Nash-Sutcliffe Efficiency Coefficient).

**Figure 6:**
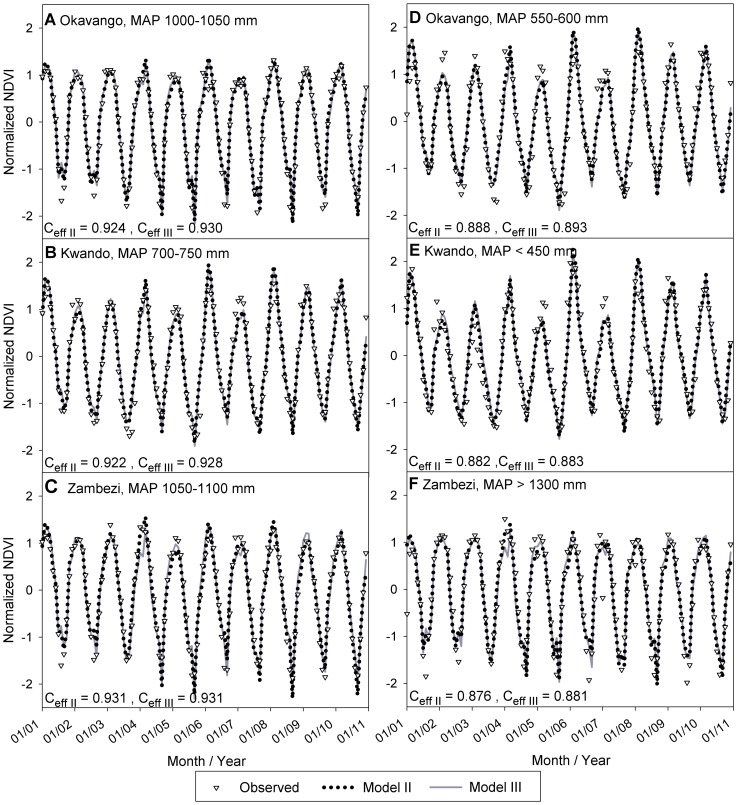
Comparison of model II and III results and measured NDVI. Best-performing (**A,B,C** in left column) and worst-performing (**D,E,F** in right column) polygons in each watershed are shown.

Finally, a multi-linear model (Model III) was developed from DFA results by removing the trend from Model II. Fig. 4 summarizes relationships between C*_eff_* and BIC for several multi-linear models explored using different combinations of CEVs (*K* = 1−6). The best Model III for each *K* is the model with the highest region-averaged C*_eff_* (symbols in Fig. 4) and best explanation of NDVI for the worst-performing polygon (lower C*_eff_* value in error bars, Fig. 4). Note that with the same set of CEVs identified in Model II (F, S, P, T), Model III only suffered a small reduction in performance (overall C*_eff_* decreased from 0.91 to 0.90; Table 2; Fig. 5), indicating the weak reliance of Model II on trend. Further addition of E met the selection criteria, yielding minimum BIC, best overall performance, and best explanation in the worst-performing polygons (Table 2 and Fig. 5). Fig. 6 presents observed and simulated NDVI time series for the best (Fig. 6**A**–**C**) and worst (Fig. 6**D**–**F**) performing polygons in each watershed. Although some extreme values are missed, both Model II and Model III closely reproduce the observed NDVI patterns across the region. Model III will be used in the ensuing discussion since it contains only explained variability (environmental covariates).

## Discussion

Understanding of environmental factors driving variance in NDVI is key in predicting how projected climate and land-use change will affect local and regional vegetation. DFA results quantify the combined spatio-temporally variable effects of the five main environmental factors driving NDVI distribution in study area over ten years, and allow addressing important questions on regional vegetation dynamics.

### How does the Relative Importance of Environmental Drivers Vary in Space?

**Figure 7:**
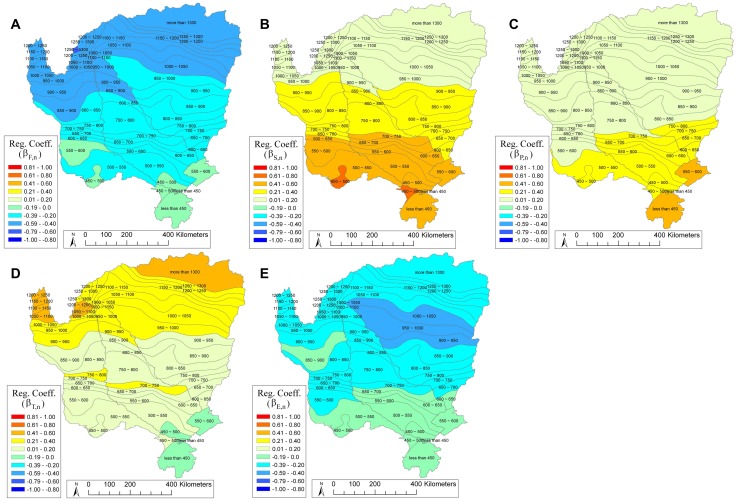
Spatial importance of the five explanatory variables in final NDVI model III. Importance is represented by the distribution of the *β_k,n_* regression coefficients (values −1 to 1) for each variable: (**A**) fire, F; (**B**) soil moisture, S; (**C**) precipitation, P; (**D**) mean temperature, T; and (**E**) potential evapotranspiration, E.

The spatial distribution of regression coefficients (*β_k,n_*) (see Table S3) represents the relative importance of the normalized explanatory variables across different regions (Fig. 7). As expected, fire (F, Fig. 7**A**) is negatively related with green vegetation, with an apparent north-south gradient in *β_F,n_* (−0.13≥ *β_F,n_* ≥ −0.61). Strongest fire effects (highest absolute *β_F,n_* values) are seen north of the 750–800 mm MAP line, suggesting that fire plays a stronger role in determining vegetation dynamics in wetter areas, likely due to greater biomass in wetter areas (and thus greater impacts of fire), and a stronger dependence of vegetation upon MAP (vis-à-vis fire) in drier regions [62].

Soil moisture (S, Fig. 7**B**) was positively related to NDVI (0.01≤*β_S,n_*≤0.64) and revealed the opposite spatial pattern to fire, with importance decreasing from south to north. The maximum *β_S,n_* found in the drier southwestern areas evidence S as a key factor in the variability of NDVI, reflecting the “hydrologic capacitance” or rainfall storage capacity of the area. In the wetter northern regions, S is less important due to more abundant MAP (*β_S,n_* reaches its minimum value where MAP>1300 mm). Area-weighted average precipitation (P, Fig. 7**C**) is positively related with NDVI (0.09≤*β*_P,n_≤0.42), with maximum *β_P,n_* values in drier areas, mainly in the Okavango and Zambezi watersheds, due to lower MAP and higher variability in these regions [63]. While S and P are inherently (physically) related, their correlation is low (*ρ* = 0.39) since moisture is further mediated by spatially variable soil characteristics. Plant-available water is a function of both S and P, and thus their inclusion improved NDVI model explanation without exceeding the VIF collinearity criterion. However, while small covariance is still present and cannot be ignored, averaging the variables at a scale that is larger than their characteristic scale of variability [64] largely reduces the small-scale correlations among variables [65]. Thus, at the larger polygon scale used the DFA, factors can effectively reflect the large-scale controls over the response variable.

Area-weighted average temperature (T, Fig. 7**D**) was also positively related with NDVI (−0.02≤*β_T,n_*≤0.48), with an apparent gradient in importance from north to south. This may be due to differences in the response of trees versus grasses to temperature variation. Timing of green-up and senescence in grasslands is dominated by C-4 plants, better adapted to high temperatures [66] and less likely to be affected by temperature (i.e. have lower *β_T,n_* values), than wooded savanna regions. Highest values in the north may reflect greater reliance on leaf-out cues based on temperature rather than photoperiod [67] in more mountainous areas, where slope and aspect affect the timing and magnitude of insolation. To our knowledge, this is the first analysis to point out the importance of temperature to variance in NDVI on savanna ecosystems.

Finally, area-weighted average potential evapotranspiration (E, Fig. 7**E**) was negatively related to NDVI (−0.42≤*β_T,n_*≤−0.01), with lowest values in the south increasing to their maximum and leveling off around 900–950 mm. Potential evapotranspiration is driven by climatic demand, taking no account of soil available water. As the seasonality of E and S are out of phase (Fig. 3), it follows that the potential evapotranspiration demand is not fully met by actual evapotranspiration. The negative relation between E and NDVI points to the potential for vegetation growth that is currently not met by the available water and becomes a limiting factor in this landscape.

**Figure 8:**
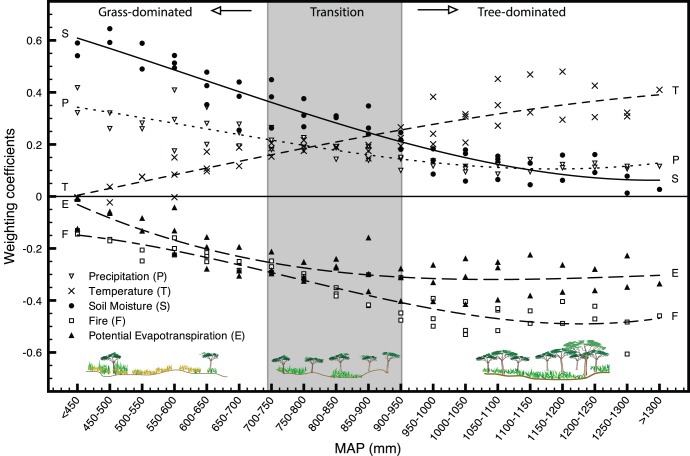
Environmental drivers of savanna ecosystems as a function of mean annual precipitation (MAP). Weight (or importance) of each driver of NDVI is described by Model III *β_k,n_* regression coefficients along MAP gradients. Lines represent the main trajectories and highlight the shift in the importance of environmental drivers along a gradient from grass- to tree-dominated savanna landscapes.

Fig. 8 summarizes the spatial organization of the importance of each environmental covariate in Model III over NDVI, highlighting the regional shift in importance in driving forces in areas receiving differing MAP and representing three distinct savanna types (from grass- to tree-dominated systems). For example, where MAP<750 mm (primarily grass-dominated savannas) we show that NDVI was most strongly influenced by soil moisture and precipitation, with much smaller effects of fire, evapotranspiration, and temperature. This supports previous studies [62], [68], that identified fire as less important than precipitation in predicting tree cover in areas with MAP<1000 mm (due to water limitation on tree growth) or >2000 mm (due to humid conditions), and Linhoss et al. [69] who identified soil moisture in the Okavango watershed as a key driver of the environmental dynamics in the Okavango Delta.

On the other hand, when MAP> ∼900 mm (where NDVI and overall biomass increase with an increasing presence of woody vegetation) markedly different patterns emerge. Fire and mean temperature come to dominate the variability in NDVI, followed in importance by evapotranspiration, while soil moisture and precipitation effects decline (vegetation is no longer as water limited). Previous studies suggest that in arid and semi-arid areas, increasing pre-season temperatures reduce water availability by increasing evaporation, thereby delaying season onset [2]. Importantly, the results summarized in Fig. 8 reveal the power of DFA for the biogeographical interpretation of spatially variable environmental effects. In particular, this analysis quantified the spatial distribution of the role of each environmental factor in driving NDVI across a large, heterogeneous domain and highlighted the transition between savanna regions dominated by S and P and those dominated by T, F, and E.

### Applicability of the Final NDVI Model

Removal of unexplained variability from the final DFM Model III yields a statistical model of NDVI based solely on a set of biophysical parameters that may provide a “first-cut” approximation of the likely response of vegetation (via NDVI) to mid- and long-term climate changes. It is important to recognize that these models are statistical, and like any forecasting models, inherently assume that the identified relationships remain consistent across a different suite of climatic variables (a problematic assumption under non-stationary climate conditions). While the sign of the identified relationships may not change, the slope and magnitude likely will. For example, while fire and NDVI would remain negatively correlated under varying climate scenarios, the importance of fire to overall NDVI could change non-linearly since it is a function of other climatic factors that influence biomass (e.g. precipitation and temperature).

The length of some remote sensing records now permit systematic quantification of the relationships between key drivers of vegetation growth and resultant vegetation cover over sufficient temporal and spatial scales to begin developing models for use as predictive future change scenarios. These data are also valuable for detecting contemporary changes not currently predicted in the modeling framework and assessing where and why models perform poorly. The approach helps improve our understanding of the dynamics of vegetative change, the role and identification of any missing drivers, and potential future impacts of increased climate variability and change. Monthly MODIS-derived vegetation indices hold considerable promise for large-scale quantification of complex vegetation-climate dynamics, and for regional analyses of landscape change related to global environmental changes [28], [70].

This research highlights the utility of coupling remote sensing and time-series tools. With the dramatic increase in global change research, this type of methodology augurs well for further development and application of spatially explicit modeling at the intersection of remote sensing and Land Change Science.

## Materials and Methods

### Study Area

The study catchments cover 681,545 km^2^ in tropical and sub-tropical southern Africa (Zambia, Angola, Namibia, and Botswana) (Fig. 1**A**). MAP ranges from <400 mm to 1400 mm yr^−1^ and is strongly correlated with latitude and elevation, with highest rainfall in the mountainous north (Fig. 1**A**). This gradient straddles a noted [3] critical threshold of 650 mm, below which precipitation is believed to dominate savanna vegetation patterns, and above which other factors such as fire and herbivory are hypothesized to play an important role. However, the cattle stocking rate in our study region is very low (<1–10 head/km^2^, compared to >250 head/km^2^ in other African savanna regions, see Appendix S2, Fig. A1) [71] and although there are a number of wildlife management areas throughout the region these are of relatively small size and low wildlife density, suggesting their effect is likely minimal at the polygon level. Thus, at the low densities present in the study region, herbivory likely reduces proportionally the amount of green vegetation in small quantities, following primarily the same seasonal cycling as plant green-up and senescence and although it could reduce slightly the magnitude of NDVI it does likely not change the temporal pattern (i.e. the variance) observed at the monthly time scale pursued in this study. Relatively low regional human population [72] reduces the effect of land use changes associated with roads and settlements, and facilitates identification of the explicit effects of climate on vegetation. Southern semi-arid areas are characterized by low MAP with high interannual variability. Both magnitude and relative reliability of MAP increases towards the north [73]. Rainfall is seasonal, with ∼87% of MAP received from November through March (Fig. 1**B**). July (mean temperature of 17.4°C) and October (mean temperature of 25.6°C) are the coldest and warmest months, respectively (Fig. 1**B**). Soils are primarily Oxisols in the north and Entisols in the south [74]. In particular, Kalahari sands characterize the majority of the area. Low topography in the south, especially in Caprivi (Namibia and northern Botswana), complicates hydrologic separation of the catchments. Historically inter-basin water flowed eastward, but the systems have remained unconnected since the late 1970s.

### Remote Sensing Data – Response and Candidate Explanatory Variables

Remote sensing data included ten years (2001–2010) of monthly NDVI data (response variable) and a suite of environmental variables used as explanatory variables (CEVs) in the DFA, including precipitation, mean temperature, maximum temperature, soil moisture, relative humidity, fire and potential evapotranspiration (Table 1).

Time series of response and explanatory variables were aggregated from pixel-scale data by extracting mean values over areas defined by different precipitation intervals for each of the three drainage basins, producing 48 individual data polygons (Fig. 1**A**). While the dominant precipitation gradient is from north (high elevation) to south (low elevation) there are also potentially significant drivers of change from east to west (topography, oceanic influence etc.), which may be better elucidated by considering three distinct drainage basins oriented along this gradient. The individual polygons (Fig. 1**A**), yielding a total of 336 time series (1 response variable and 6 CEVs in each of 48 polygons) comprised of 120 monthly average values.

#### Vegetation: NDVI

MODIS NDVI data (MOD13A3) were applied as the response (dependent) variable (Table 1) since NDVI has been closely linked with green cover, vegetation primary production, and phenology of savanna systems [33], [75]–[77]. MODIS provides monthly NDVI data at a 1-kilometer spatial resolution in the sinusoidal projection. Grids contaminated by clouds and those with average growing season NDVI less than 0.1 were excluded from analysis. Future research will evaluate the suitability of these models for earlier data series (i.e. AVHRR data), but we wished to avoid potentially confusing the time-series by incorporating two distinct (spatial, temporal, spectral and radiometric) datasets into one analysis.

#### Fire data

The MODIS Thermal Anomalies/Fire data [78], were applied as an explanatory variable of fire frequency (Table 1). This is an 8-day fire-mask composite image at 1-kilometer resolution in the Sinusoidal projection. Numbers of fire pixels within each precipitation interval were calculated at an 8-day scale, and the number of fire pixels summarized to characterize the monthly fire frequency within each polygon.

#### Climate: precipitation, temperature, potential evapotranspiration, and soil moisture

We utilized datasets of monthly precipitation (2001–2010) and monthly mean and maximum air temperature (2001–2010), which have a spatial resolution of 0.5 degree by 0.5 degree with grid nodes centered on 0.25 degree (Table 1). These improve upon previous global mean monthly datasets using a refined Shepard interpolation algorithm and increased numbers of neighboring station points [79]. Based on grid nodes included in the three catchments we interpolated continuous surfaces with 1-kilometer spatial resolution using inverse distance weighting. Potential evapotranspiration impacts water availability to vegetation and therefore influences savanna ecosystems considerably. NCEP-DOE Reanalysis II global potential evapotranspiration data (Table 1) was used to represent environmental demand for evapotranspiration. Irregular Gaussian grids were converted to continuous surfaces of 1-kilometer spatial resolution via inverse distance interpolation method in ArcGIS.

Soil moisture is an integrated factor exerting the dominant control on the spatial distribution of trees, shrubs, and grasses. The Climate Prediction Center (CPC) global monthly soil moisture data (Table 1) provides monthly values at a 0.5-degree spatial resolution with grid nodes centered on 0.25 degree, based on a one-layer “bucket” water balance model, which uses CPC monthly global precipitation data and monthly temperatures as input fields [80].

### Dynamic Factor Analysis (DFA)

DFA is a statistical explanatory tool built upon common patterns among, and interactions between, response and explanatory time series. Thus, no *a priori* understanding of interactions between response (NDVI) and explanatory variables (e.g. precipitation, fire etc.) is required [53]. It is inherently a structural time series technique [40] that models temporal variation in observed data series (response variable) as linear combinations of one or more common trends (representing *unexplained variability*), zero or more external explanatory variables (representing *explained variability*), a constant intercept parameter, and noise [41], [81], [82] as:
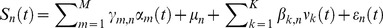
(1)

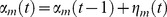
(2)where *S_n_*(*t*) is a vector containing the set of *N* response variables (*n = 1,N*); *α_m_*(*t*) is a vector containing the *M* common trends (*m = 1,M*); *γ_m,n_* are factor loadings or weighting coefficients, which indicate the importance of each of the common trends; *µ_n_* is a constant level parameter; *υ_k_*(*t*) is a vector containing the *K* explanatory variables (*k = 0,K*); and *β_k,n_* are regression coefficients indicating the importance of each of the explanatory variable. Here, *S_n_* represents the 48 NDVI time series (one from each polygon in Fig. 1**A**). Terms *ε_n_*(*t*) and *η_m_*(*t*) are independent Gaussian noise with zero mean and unknown diagonal or symmetric/non-diagonal covariance matrix. In order to produce models with the smallest number of common trends, non-diagonal error covariance matrices were used [83].

As with other modeling tools, DFA aims to balance goodness-of-fit and model parsimony by developing different dynamic factor models (DFM). We assessed DFM performance using the Nash & Sutcliffe [84] coefficient of efficiency (*C_eff_*) with significance test [85] and Bayesian Information Criterion (BIC) [86]. The best DFM minimizes numbers of common trends required to achieve the best fit as determined by C*_eff_* and/or BIC. Appropriate explanatory variables may help improve the model, identify which environmental factors (if any) affect the response variables, and quantify the spatial distribution of the importance of each factor. To compare the relative importance of common trends and explanatory variables across response variables, all response and explanatory variables were normalized (mean subtracted, divided by standard deviation) prior to analysis [43], [81]. Canonical correlation coefficients (*ρ_m_*_,*n*_) quantified cross-correlation between response variables and common trends, with values of *ρ_m_*_,*n*_>0.5 indicating high correlations. DFA was implemented using Brodgar software (v2.7.2, Highland Statistics Ltd., UK).

### Dimension Reduction of Candidate Explanatory Variables

Nine available candidate explanatory variables (CEV) (Table A1 in Appendix S1) were initially considered, consisting of monthly times series of precipitation, mean temperature, minimum temperature, maximum temperature, soil moisture, fire, relative humidity, actual evapotranspiration and potential evapotranspiration. The variance inflation factor (VIF) was used to quantify the severity of collinearity of each set of CEVs [83]. Combinations of CEVs with VIF>10 were excluded from the analyses [83], [87], [88]. Prior to performing the DFA, with the goal of identifying the most useful representation of this large suite of potential explanatory variables, we explored the reduction of the 48 polygon-based time series for each of the non-collinear variables. An initial dimension reduction of the large suite of environmental covariate time series was performed by carrying out a preliminary DFA on the CEVs. In this approach, each CEV was considered as a set of 48 response variables and was analyzed to assess the feasibility of representing the CEV with a much-reduced set of variables. Two DFMs were explored: 1) a base-line model consisting of only common trends without explanatory variables, and 2) a model consisting of one or more common trends and a single explanatory variable calculated as the area-weighted average of the 48 response variables. In the latter, the area-weighted average represents the domain-wide “average” CEV, while the trends represent remaining unexplained variation (or “anomalies”) from this average. In both models, trends and variables were added until a minimum in BIC was achieved. This approach reduced each CEV from an initial set of 48 time series to a smaller set of time series for exploration of NDVI through DFA.

### NDVI Analysis Procedure

DFA of NDVI was performed in three steps. DFMs were developed using an increasing number of common trends without CEVs until satisfactory model performance was achieved according to goodness-of-fit indicators [82], referred to as *Model I*. Next, combinations of CEVs were incorporated to create *Model II* aimed at reducing unexplained variability (fewer number and reduced weight of common trends, represented by lower factor loadings) and improve description of NDVI (goodness-of-fit metrics). Finally, *Model III* was explored by removing unexplained variability (common trends) when optimizing a multi-linear model consisting of the CEVs identified in Model II. Multiple regression code in Matlab (v2012a, The MathWorks, Inc., USA) was used in the optimization process. Model III permitted refining of selection of the most important CEVs, since inclusion of trends in Model II may mask effects of important explanatory variables of the multivariate model [52]. When selecting the “best” model, we adopted a multi-criteria objective consisting of: a) model adequacy (minimizing BIC); b) global model goodness-of-fit (maximizing C*_eff_*); c) improvement in C*_eff_* for the worst-performing polygons (minimizing range in C*_eff_*); and d) reduction in importance of common trends (i.e. minimizing *ρ_m,n_*).

## Conclusions

Dynamic factor analysis was applied to study variation in NDVI across three large watersheds in southern Africa and to identify factors driving observed variations in savanna vegetation across physiographic gradients. The analysis framework allowed a dimension reduction of the large suite of candidate explanatory variables, identifying area-weighted domain averages of fire, soil moisture, precipitation, temperature, and potential evapotranspitarion as important environmental factors driving NDVI, with negligible importance of unexplained variability.

This novel approach permitted analysis of shared spatial effects of potentially important environmental variables on NDVI. In contrast, most previous studies of NDVI in southern Africa have focused on individual relationships between NDVI and one or two explanatory variables [2], [3], highlighting some of trends relating to individual factors but ignoring a suite of variables acting in concert. Our results support the importance of the spatial distribution of soil moisture [89], and precipitation and fire [4], [62], [63] on NDVI, but also points to other overlooked effects of temperature and potential evapotranspiration, particularly in regions with MAP> ∼950 mm. The spatial distribution of environmental covariates evinces transitional importance over NDVI from grass-dominated regions with MAP<750 mm (dominated by precipitation and soil moisture) to tree-dominated regions with MAP>950 mm (dominated by fire, potential evapotranspiration, and temperature). Through the use of such models we can now better evaluate and understand landscape level changes in vegetation amounts. This improves characterization of the landscape upon which examinations of change mechanisms and drivers are based, is of unparalleled use to managers and researchers alike, and constitutes a powerful tool. The potential power and utility of such techniques permeate biogeography, ecology, land change and remote sensing studies, especially in the context of global environmental change.

## Supporting Information

Table S1 **Initial dimensional reduction of candidate explanatory variables.**
(DOCX)

Table S2 **Dimensional reduction of Normalized Difference Vegetation Index (NDVI).**
(DOCX)

Table S3 **Spatial distribution of weighting coefficients for model III.**
(DOCX)

Appendix S1 **Collinearity test of explanatory variables.**
(DOCX)

Appendix S2 **Herbivory in the study area.**
(DOCX)
