# Supplementary material for: Beyond Precipitation: Physiographic Gradients Dictate the Relative Importance of Environmental Drivers on Savanna Vegetation
Source: PLoS One. 2013 Aug 30;8(8):e72348. doi: 10.1371/journal.pone.0072348 (PMC3758306; doi:10.1371/journal.pone.0072348)
Supplement: Table S1 — Initial dimensional reduction of candidate explanatory variables. (DOCX) [file pone.0072348.s001.docx]

**Table S1.** Initial dimensional reduction of candidate explanatory variables. Response variables are time series of the spatially distributed environmental variables (48 polygons in Fig. 1) and explanatory variables are the weighted averages for the region. Numbers in bold represent selected models.

|  |  | Number of Trends (M) | | | | | | | |
| --- | --- | --- | --- | --- | --- | --- | --- | --- | --- |
| Response  variable | Explanatory  variable | 1 | 2 | 3 | 4 | 1 | 2 | 3 | 4 |
|  |  | BIC^*^ | | | | C_eff_^**^ | | | |
| Precipitation | - | -26412 | -26131 | -25851 | -25589 | 0.80  (0.61-0.88) | 0.81  (0.63-0.89) | 0.81  (0.61-0.87) | 0.83  (0.65-0.89) |
|  | **P** | **-28902** | -28603 | -28291 | -28025 | **0.94**  **(0.80-0.99)** | 0.95  (0.80-0.99) | 0.95  (0.80-1.00) | 0.95  (0.82-0.99) |
| Mean Temperature | - | -24001 | -23769 | -23519 | -23275 | 0.70  (0.52-0.83) | 0.68  (0.53-0.79) | 0.68  (0.53-0.82) | 0.66  (0.51-0.80) |
|  | **T** | **-26288** | -26028 | -25759 | -25463 | **0.96**  **(0.85-1.00)** | 0.97  (0.88-1.00) | 0.97  (0.87-1.00) | 0.97  (0.87-1.00) |
| Max. Temperature | - | -28972 | -28735 | -28555 | -28259 | 0.73  (0.47-0.86) | 0.79  (0.66-0.88) | 0.88  (0.82-0.93) | 0.89  (0.82-0.93) |
|  | **M** | **-30913** | -30707 | -30479 | -30256 | **0.95**  **(0.82-0.99)** | 0.93  (0.80-0.99) | 0.95  (0.82-0.99) | 0.97  (0.94-0.99) |
| Soil Moisture | - | -22278 | -22189 | -22091 | -22015 | 0.73  (0.42-0.83) | 0.75  (0.38-0.87) | 0.83  (0.76-0.89) | 0.79  (0.66-0.87) |
|  | **S** | **-23453** | -23351 | -23296 | -23147 | **0.90**  **(0.64-0.98)** | 0.90  (0.67-0.98) | 0.93  (0.82-0.98) | 0.95  (0.85-0.98) |
| Fire | - | 11513 | 11743 | 11995 | 12238 | 0.56  (0.01-0.86) | 0.58  (0.00-0.87) | 0.57  (0.00-0.89) | 0.58  (0.02-0.88) |
|  | **F** | **10000** | 10245 | 10501 | 10760 | **0.74**  **(0.02-0.93)** | 0.75  (0.02-0.94) | 0.75  (0.03-0.94) | 0.76  (0.02-0.94) |
| Potential  Evapotranspiration | - | -29846 | -29580 | -29334 | -29086 | 0.70  (0.38-0.83) | 0.82  (0.74-0.86) | 0.85  (0.81-0.88) | 0.84  (0.80-0.89) |
|  | **E** | **-31236** | -30986 | -30702 | -30446 | **0.93**  **(0.82-0.99)** | 0.91  (0.79-0.99) | 0.94  (0.83-0.99) | 0.95  (0.88-0.99) |

^**^ BIC: Bayesian Information Criterion; ^**^ C_eff_: Nash-Sutcliffe coefficient of efficiency. Values presented are area weighted-averages for the region (range in parenthesis).
