# Supplementary material for: Beyond Precipitation: Physiographic Gradients Dictate the Relative Importance of Environmental Drivers on Savanna Vegetation
Source: PLoS One. 2013 Aug 30;8(8):e72348. doi: 10.1371/journal.pone.0072348 (PMC3758306; doi:10.1371/journal.pone.0072348)
Supplement: Table S2 — Dimensional reduction of Normalized Difference Vegetation Index (NDVI). (DOCX) [file pone.0072348.s002.docx]

**Table S2.** Dimensional reduction of Normalized Difference Vegetation Index (NDVI). BIC, C_eff_ for each model II and III (0 trends). Numbers in bold represent selected model. Explanatory variables are area-weighted values for the region: precipitation (P), mean temperature (T), maximum temperature (M), soil moisture (S), fire (F) and potential evapotranspiration (E). Explanatory variables followed by a “1” indicate the trend (“anomaly”) fitted to the explanatory variables in the initial dimension reduction.

|  | Number of Trends (M) | | | | | | | | | | |  |
| --- | --- | --- | --- | --- | --- | --- | --- | --- | --- | --- | --- | --- |
|  | BIC^a^ | | | | | C_eff_^b^ | | | | | *ρ_1,n_*^c^ |  |
| Expl.  Var. | 0 | 1 | 2 | 3 | 4 | 0 | 1 | 2 | 3 | 4 | 1 |  |
| P | -3848 | -1683 | -1504 | -1308 | -1137 | 0.55 (0.44-0.63) | 0.86 (0.70-0.94) | 0.87 (0.72-0.94) | 0.87 (0.72-0.93) | 0.87 (0.73-0.93) | 0.73 (0.53-0.85) | |
| T | 364 | -1712 | -1566 | -1373 | -1198 | 0.07 (0.00-0.27) | 0.91 (0.79-0.96) | 0.91 (0.79-0.95) | 0.91 (0.79-0.96) | 0.91 (0.79-0.95) | 0.94 (0.84-0.98) | |
| M | -1868 | -1704 | -1521 | -1316 | -1155 | 0.37 (0.13-0.52) | 0.91 (0.79-0.96) | 0.90 (0.78-0.94) | 0.90 (0.80-0.94) | 0.89 (0.80-0.92) | 0.86 (0.77-0.92) | |
| S | -3389 | -1807 | -1626 | -1404 | -1187 | 0.52 (0.25-0.77) | 0.89 (0.79-0.93) | 0.89 (0.78-0.94) | 0.91 (0.80-0.95) | 0.91 (0.81-0.95) | 0.74 (0.55-0.88) | |
| F | -6094 | -1706 | -1519 | -1329 | -1163 | 0.70 (0.51-0.81) | 0.87 (0.74-0.92) | 0.88 (0.78-0.93) | 0.88 (0.81-0.92) | 0.88 (0.78-0.92) | 0.77 (0.60-0.90) | |
| E | -4212 | -1758 | -1592 | -1398 | -1239 | 0.58 (0.32-0.69) | 0.88 (0.73-0.95) | 0.89 (0.78-0.93) | 0.92 (0.85-0.96) | 0.91 (0.82-0.96) | 0.76 (0.70-0.82) | |
| P T | -3850 | -1502 | -1356 | -1167 | -988 | 0.58 (0.51-0.66) | 0.87 (0.78-0.92) | 0.88 (0.78-0.92) | 0.89 (0.77-0.94) | 0.89 (0.78-0.94) | 0.76 (0.63-0.83) | |
| P S | -7455 | -1655 | -1460 | -1274 | -1090 | 0.78 (0.62-0.89) | 0.84 (0.71-0.90) | 0.84 (0.69-0.92) | 0.83 (0.68-0.93) | 0.84 (0.69-0.93) | 0.48 (0.26-0.58) | |
| P F | -7451 | -1519 | -1329 | -1147 | -954 | 0.78 (0.59-0.87) | 0.89 (0.81-0.93) | 0.88 (0.75-0.93) | 0.91 (0.83-0.94) | 0.91 (0.83-0.94) | 0.60 (0.34-0.80) | |
| P E | -7163 | -1568 | -1392 | -1219 | -1002 | 0.77 (0.65-0.84) | 0.85 (0.69-0.92) | 0.86 (0.73-0.91) | 0.85 (0.72-0.92) | 0.85 (0.73-0.90) | 0.60 (0.47-0.70) | |
| T S | -5164 | -1672 | -1470 | -1291 | -1114 | 0.67 (0.51-0.82) | 0.81 (0.68-0.88) | 0.84 (0.68-0.92) | 0.85 (0.71-0.92) | 0.87 (0.73-0.93) | 0.59 (0.40-0.70) | |
| T F | -6122 | -1566 | -1404 | -1229 | -1028 | 0.72 (0.51-0.86) | 0.90 (0.84-0.93) | 0.91 (0.86-0.95) | 0.89 (0.83-0.93) | 0.90 (0.84-0.93) | 0.78 (0.60-0.92) | |
| T E | -8478 | -1724 | -1540 | -1356 | -874 | 0.81 (0.72-0.86) | 0.87 (0.77-0.91) | 0.87 (0.77-0.91) | 0.87 (0.77-0.90) | 0.90 (0.81-0.94) | 0.36 (0.21-0.52) | |
| S F | -8719 | -1648 | -1465 | -1304 | -1108 | 0.82 (0.70-0.87) | 0.91 (0.84-0.94) | 0.89 (0.83-0.94) | 0.90 (0.81-0.93) | 0.89 (0.81-0.93) | 0.61 (0.45-0.71) | |
| S E | -4337 | -1585 | -1398 | -1212 | -1027 | 0.62 (0.33-0.80) | 0.92 (0.83-0.97) | 0.93 (0.83-0.96) | 0.93 (0.84-0.96) | 0.93 (0.83-0.96) | 0.75 (0.60-0.88) | |
| M E | -3887 | -1489 | -1326 | -1124 | -907 | 0.59 (0.34-0.69) | 0.91 (0.82-0.96) | 0.91 (0.81-0.95) | 0.89 (0.80-0.94) | 0.88 (0.78-0.92) | 0.78 (0.68-0.86) | |
| M F | -7086 | -1545 | -1389 | -1216 | -974 | 0.76 (0.68-0.83) | 0.88 (0.78-0.93) | 0.86 (0.78-0.91) | 0.84 (0.76-0.89) | 0.85 (0.77-0.90) | 0.71 (0.62-0.79) | |
| M S | -3082 | -1555 | -1354 | -1169 | -961 | 0.52 (0.25-0.78) | 0.91 (0.82-0.96) | 0.88 (0.78-0.92) | 0.89 (0.78-0.94) | 0.89 (0.78-0.94) | 0.77 (0.60-0.90) | |
| T M | -4202 | -1580 | -1407 | -1221 | -1013 | 0.61 (0.54-0.65) | 0.87 (0.75-0.94) | 0.86 (0.75-0.91) | 0.88 (0.75-0.94) | 0.88 (0.76-0.94) | 0.64 (0.57-0.69) | |
| P M | -5811 | -1533 | -1326 | -1181 | -976 | 0.70 (0.60-0.78) | 0.84 (0.71-0.90) | 0.82 (0.69-0.89) | 0.80 (0.69-0.86) | 0.84 (0.71-0.90) | 0.65 (0.54-0.70) | |
| F E | -7519 | -1609 | -1430 | -1245 | -1039 | 0.78 (0.67-0.86) | 0.87 (0.73-0.92) | 0.89 (0.80-0.93) | 0.88 (0.79-0.92) | 0.89 (0.79-0.93) | 0.58 (0.46-0.70) | |
| P T S | -7491 | -1508 | -1323 | -1110 | -913 | 0.79 (0.65-0.89) | 0.83 (0.70-0.90) | 0.84 (0.70-0.90) | 0.84 (0.71-0.90) | 0.85 (0.69-0.94) | 0.33 (0.11-0.44) | |
| P T F | -7598 | -1339 | -1180 | -996 | -799 | 0.80 (0.65-0.88) | 0.90 (0.85-0.93) | 0.92 (0.87-0.94) | 0.91 (0.87-0.94) | 0.91 (0.87-0.93) | 0.63 (0.42-0.78) | |
| P T E | -8629 | -1443 | 1245 | -1043 | -852 | 0.83 (0.77-0.88) | 0.88 (0.77-0.92) | 0.87 (0.77-0.92) | 0.88 (0.77-0.92) | 0.88 (0.78-0.92) | 0.33 (0.17-0.50) | |
| P S F | -10975 | -1510 | -1312 | -1121 | -911 | 0.89 (0.81-0.92) | 0.90 (0.83-0.94) | 0.90 (0.83-0.94) | 0.91 (0.83-0.94) | 0.90 (0.83-0.93) | 0.26 (0.06-0.35) | |
| P S E | -7813 | -1410 | -1155 | -1021 | -850 | 0.81 (0.65-0.90) | 0.86 (0.72-0.92) | 0.87 (0.73-0.93) | 0.86 (0.72-0.94) | 0.87 (0.71-0.95) | 0.50 (0.28-0.56) | |
| P F E | -9190 | -1432 | -1320 | -1261 | -1015 | 0.85 (0.76-0.90) | 0.88 (0.81-0.92) | 0.88 (0.81-0.92) | 0.89 (0.83-0.92) | 0.89 (0.83-0.92) | 0.39 (0.21-0.56) | |
| T S F | -10180 | -1522 | -1372 | -1174 | -953 | 0.87 (0.82-0.91) | 0.90 (0.85-0.93) | 0.90 (0.85-0.93) | 0.89 (0.84-0.92) | 0.91 (0.88-0.94) | 0.34 (0.28-0.41) | |
| T S E | -9044 | -1534 | -1348 | -1157 | -938 | 0.84 (0.75-0.88) | 0.88 (0.78-0.94) | 0.88 (0.78-0.93) | 0.89 (0.78-0.94) | 0.89 (0.78-0.95) | 0.27 (0.16-0.41) | |
| T F E | -9887 | -1526 | -1325 | -1129 | -957 | 0.86 (0.73-0.92) | 0.91 (0.86-0.92) | 0.91 (0.86-0.93) | 0.90 (0.85-0.93) | 0.90 (0.84-0.93) | 0.30 (0.14-0.48) | |
| M F E | -7253 | -1311 | -1156 | -961 | -750 | 0.79 (0.70-0.87) | 0.89 (0.78-0.94) | 0.87 (0.79-0.91) | 0.88 (0.80-0.92) | 0.85 (0.75-0.91) | 0.65 (0.56-0.73) | |
| M S E | -4061 | -1314 | -1102 | -916 | -737 | 0.63 (0.36-0.80) | 0.92 (0.83-0.96) | 0.90 (0.80-0.94) | 0.91 (0.80-0.95) | 0.91 (0.81-0.95) | 0.72 (0.57-0.84) | |
| M S F | -8400 | -1425 | -1258 | -1038 | -882 | 0.82 (0.72-0.87) | 0.90 (0.84-0.94) | 0.90 (0.83-0.93) | 0.89 (0.82-0.93) | 0.88 (0.80-0.91) | 0.51 (0.32-0.63) | |
| T M E | -8415 | -1456 | -1253 | -1007 | -826 | 0.82 (0.75-0.87) | 0.87 (0.76-0.91) | 0.87 (0.76-0.91) | 0.85 (0.76-0.89) | 0.87 (0.77-0.90) | 0.28 (0.15-0.46) | |
| T M F | -8307 | -1443 | -1288 | -1084 | -893 | 0.82 (0.69-0.89) | 0.88 (0.84-0.91) | 0.88 (0.83-0.92) | 0.89 (0.84-0.92) | 0.89 (0.83-0.92) | 0.42 (0.28-0.57) | |
| T M S | -5714 | -1415 | -1239 | -1055 | -849 | 0.72 (0.58-0.84) | 0.87 (0.74-0.92) | 0.87 (0.73-0.94) | 0.85 (0.71-0.93) | 0.83 (0.68-0.93) | 0.55 (0.38-0.64) | |
| P M E | -6901 | -1318 | -1131 | -939 | -742 | 0.77 (0.65-0.84) | 0.84 (0.69-0.92) | 0.87 (0.75-0.92) | 0.85 (0.74-0.91) | 0.85 (0.73-0.91) | 0.55 (0.43-0.64) | |
| P M F | -8894 | -1390 | -1222 | -1008 | -842 | 0.84 (0.76-0.88) | 0.88 (0.81-0.91) | 0.88 (0.82-0.92) | 0.88 (0.83-0.91) | 0.88 (0.83-0.91) | 0.44 (0.31-0.57) | |
| P M S | -7171 | -1419 | -1215 | -1014 | -810 | 0.78 (0.62-0.90) | 0.84 (0.69-0.90) | 0.85 (0.71-0.90) | 0.86 (0.71-0.94) | 0.85 (0.70-0.93) | 0.44 (0.22-0.54) | |
| P T M | -5890 | -1333 | -1181 | -985 | -754 | 0.73 (0.66-0.79) | 0.86 (0.75-0.91) | 0.86 (0.75-0.91) | 0.86 (0.75-0.90) | 0.85 (0.74-0.90) | 0.57 (0.49-0.62) | |
| S F E | -8438 | -1414 | -1223 | -1057 | -931 | 0.83 (0.71-0.88) | 0.90 (0.84-0.95) | 0.90 (0.82-0.94) | 0.90 (0.81-0.94) | 0.90 (0.83-0.94) | 0.54 (0.37-0.64) | |
| **P T S F** | -11391 | **-1376** | -1138 | -937 | -764 | 0.90 (0.86-0.92) | **0.91 (0.88-0.93)** | 0.92 (0.88-0.94) | 0.93 (0.88-0.95) | 0.93 (0.88-0.95) | **0.09 (-0.09-0.20)** | |
| P T S E | -9476 | -1261 | -1056 | -783 | -648 | 0.86 (0.77-0.91) | 0.88 (0.77-0.94) | 0.89 (0.78-0.95) | 0.89 (0.78-0.95) | 0.89 (0.78-0.95) | 0.16 (0.02-0.29) | |
| P T F E | -10005 | -1245 | -1110 | -841 | -707 | 0.88 (0.77-0.92) | 0.91 (0.87-0.93) | 0.91 (0.85-0.93) | 0.90 (0.82-0.93) | 0.91 (0.88-0.93) | 0.30 (0.13-0.48) | |
| P S F E | -10730 | -1309 | -1084 | -900 | -713 | 0.89 (0.82-0.92) | 0.90 (0.84-0.93) | 0.91 (0.84-0.94) | 0.91 (0.84-0.94) | 0.92 (0.85-0.95) | 0.17 (-0.04-0.27) | |
| M S F E | -8097 | -1141 | -974 | -797 | -600 | 0.83 (0.73-0.88) | 0.91 (0.85-0.94) | 0.91 (0.85-0.94) | 0.90 (0.82-0.93) | 0.90 (0.83-0.93) | 0.53 (0.38-0.63) | |
| T M F E | -9993 | -1263 | -1077 | -877 | -677 | 0.88 (0.77-0.92) | 0.91 (0.86-0.92) | 0.91 (0.86-0.93) | 0.91 (0.86-0.93) | 0.91 (0.86-0.93) | 0.24 (0.08-0.42) | |
| T M S E | -8725 | -1238 | -1033 | -833 | -621 | 0.85 (0.76-0.88) | 0.88 (0.78-0.94) | 0.88 (0.77-0.94) | 0.88 (0.77-0.91) | 0.88 (0.77-0.93) | 0.28 (0.17-0.41) | |
| T M S F | -10204 | -1286 | -1130 | -904 | -722 | 0.88 (0.83-0.91) | 0.90 (0.84-0.94) | 0.91 (0.87-0.94) | 0.91 (0.87-0.94) | 0.91 (0.87-0.93) | 0.27 (0.15-0.35) | |
| P M F E | -9048 | -1128 | -978 | -766 | -559 | 0.85 (0.79-0.90) | 0.89 (0.81-0.92) | 0.89 (0.83-0.92) | 0.89 (0.82-0.92) | 0.88 (0.82-0.92) | 0.45 (0.31-0.59) | |
| P M S E | -7487 | -1145 | -931 | -751 | -577 | 0.81 (0.65-0.90) | 0.86 (0.73-0.91) | 0.87 (0.73-0.94) | 0.87 (0.73-0.93) | 0.87 (0.74-0.93) | 0.46 (0.23-0.53) | |
| P M S F | -10713 | -1277 | -1092 | -857 | -688 | 0.89 (0.82-0.92) | 0.91 (0.84-0.94) | 0.91 (0.84-0.94) | 0.92 (0.86-0.95) | 0.92 (0.86-0.95) | 0.22 (0.03-0.30) | |
| P T M E | -8549 | -1180 | -985 | -792 | -528 | 0.84 (0.77-0.88) | 0.87 (0.77-0.91) | 0.87 (0.77-0.91) | 0.87 (0.77-0.91) | 0.88 (0.78-0.92) | 0.30 (0.16-0.48) | |
| P T M F | -9033 | -1173 | -1020 | -856 | -615 | 0.85 (0.76-0.91) | 0.90 (0.84-0.93) | 0.90 (0.86-0.92) | 0.90 (0.85-0.92) | 0.88 (0.80-0.91) | 0.41 (0.28-0.52) | |
| P T M S | -7450 | -1215 | -1015 | -764 | -612 | 0.81 (0.67-0.90) | 0.86 (0.73-0.90) | 0.87 (0.73-0.94) | 0.85 (0.72-0.91) | 0.87 (0.73-0.93) | 0.39 (0.18-0.50) | |
| T S F E | -11184 | -1322 | -1109 | -909 | -753 | 0.90 (0.84-0.93) | 0.92 (0.88-0.94) | 0.92 (0.88-0.94) | 0.91 (0.85-0.94) | 0.92 (0.88-0.94) | 0.20 (0.07-0.35) | |
| T M S F E | -10898 | -1038 | -861 | -586 | -459 | 0.90 (0.84-0.93) | 0.92 (0.88-0.94) | 0.92 (0.88-0.94) | 0.91 (0.85-0.94) | 0.92 (0.89-0.94) | 0.19 (0.06-0.33) | |
| P M S F E | -10401 | -1020 | -814 | -621 | -423 | 0.89 (0.82-0.92) | 0.91 (0.84-0.94) | 0.91 (0.84-0.94) | 0.91 (0.84-0.94) | 0.91 (0.85-0.93) | 0.22 (0.02-0.31) | |
| P T M F E | -10091 | -979 | -759 | -585 | -351 | 0.89 (0.79-0.93) | 0.91 (0.87-0.93) | 0.91 (0.88-0.93) | 0.90 (0.84-0.93) | 0.91 (0.87-0.93) | 0.28 (0.12-0.46) | |
| P T M S E | -9122 | -953 | -743 | -548 | -365 | 0.87 (0.77-0.91) | 0.89 (0.78-0.94) | 0.88 (0.77-0.93) | 0.89 (0.77-0.94) | 0.89 (0.77-0.96) | 0.28 (0.17-0.37) | |
| P T M S F | -11186 | -1083 | -877 | -686 | -525 | 0.91 (0.86-0.92) | 0.92 (0.88-0.94) | 0.92 (0.88-0.94) | 0.93 (0.88-0.95) | 0.92 (0.88-0.94) | 0.15 (-0.03-0.26) | |
| **P T S F E** | **-11719** | -1048 | -869 | -682 | -473 | **0.91 (0.88-0.93)** | 0.93 (0.88-0.95) | 0.93 (0.88-0.95) | 0.93 (0.88-0.95) | 0.93 (0.88-0.95) | **0.13 (-0.01-0.27)** | |
| P T M S F E | -11396 | -756 | -561 | -372 | -200 | 0.92 (0.88-0.93) | 0.93 (0.88-0.95) | 0.93 (0.88-0.95) | 0.93 (0.88-0.95) | 0.93 (0.88-0.95) | 0.15 (0.03-0.26) | |
| P1 P | -3495 | -1416 | -1237 | -1051 | -868 | 0.56 (0.46-0.63) | 0.85 (0.70-0.93) | 0.87 (0.73-0.93) | 0.86 (0.73-0.92) | 0.86 (0.72-0.92) | 0.70 (0.49-0.81) | |
| P1 T | 692 | -1431 | -1279 | -1072 | -929 | 0.08 (0.02-0.28) | 0.91 (0.80-0.96) | 0.91 (0.80-0.96) | 0.91 (0.79-0.95) | 0.90 (0.79-0.95) | 0.93 (0.83-0.98) | |
| P1 S | -3026 | -1526 | -1337 | -1141 | -949 | 0.52 (0.26-0.77) | 0.90 (0.81-0.94) | 0.91 (0.82-0.95) | 0.90 (0.79-0.95) | 0.91 (0.80-0.96) | 0.78 (0.62-0.90) | |
| P1 F | -5800 | -1408 | -1240 | -1047 | -866 | 0.70 (0.53-0.81) | 0.89 (0.79-0.95) | 0.88 (0.78-0.93) | 0.88 (0.78-0.93) | 0.89 (0.79-0.93) | 0.83 (0.71-0.90) | |
| P1 E | -3844 | -1476 | -1309 | -1125 | -947 | 0.58 (0.32-0.70) | 0.90 (0.77-0.96) | 0.88 (0.77-0.93) | 0.92 (0.83-0.96) | 0.92 (0.83-0.96) | 0.76 (0.68-0.84) | |
| T1 P | -3497 | -1411 | -1218 | -1017 | -832 | 0.56 (0.46-0.64) | 0.86 (0.71-0.93) | 0.87 (0.72-0.94) | 0.88 (0.73-0.94) | 0.86 (0.71-0.93) | 0.70 (0.50-0.81) | |
| T1 T | 595 | -1442 | -1286 | -1094 | -1266 | 0.10 (0.04-0.28) | 0.89 (0.77-0.95) | 0.89 (0.77-0.94) | 0.91 (0.79-0.95) | 0.90 (0.78-0.96) | 0.91 (0.82-0.96) | |
| T1 S | -3003 | -1532 | -1324 | -1152 | -939 | 0.52 (0.25-0.78) | 0.88 (0.78-0.94) | 0.90 (0.80-0.95) | 0.90 (0.80-0.95) | 0.89 (0.78-0.95) | 0.70 (0.50-0.84) | |
| T1 F | -5812 | -1432 | -1223 | -1020 | -820 | 0.70 (0.53-0.81) | 0.88 (0.75-0.93) | 0.88 (0.77-0.93) | 0.87 (0.78-0.91) | 0.88 (0.83-0.91) | 0.79 (0.63-0.89) | |
| T1 E | -4041 | -1481 | -1297 | -1083 | -913 | 0.60 (0.32-0.72) | 0.89 (0.75-0.95) | 0.88 (0.77-0.92) | 0.90 (0.83-0.94) | 0.92 (0.83-0.95) | 0.75 (0.66-0.83) | |
| S1 P | -3474 | -1473 | - | - | - | 0.56 (0.46-0.63) | 0.88 (0.74-0.94) | - | - | - | 0.77 (0.60-0.85) | |
| S1 T | 726 | -1496 | - | - | - | 0.08 (0.02-0.28) | 0.91 (0.79-0.96) | - | - | - | 0.93 (0.84-0.98) | |
| S1 S | -3113 | -1575 | - | - | - | 0.53 (0.26-0.77) | 0.91 (0.82-0.95) | - | - | - | 0.78 (0.61-0.90) | |
| S1 F | -5742 | -1489 | - | - | - | 0.70 (0.52-0.81) | 0.87 (0.74-0.92) | - | - | - | 0.78 (0.61-0.90) | |
| S1 E | -3849 | -1540 | - | - | - | 0.58 (0.32-0.69) | 0.90 (0.77-0.95) | - | - | - | 0.76 (0.67-0.84) | |
| F1 P | -3480 | -1350 | - | - | - | 0.56 (0.44-0.64) | 0.86 (0.71-0.94) | - | - | - | 0.74 (0.55-0.85) | |
| F1 T | 767 | -1379 | - | - | - | 0.07 (0.00-0.28) | 0.91 (0.80-0.96) | - | - | - | 0.94 (0.84-0.98) | |
| F1 S | -2979 | -1472 | - | - | - | 0.52 (0.25-0.77) | 0.89 (0.81-0.93) | - | - | - | 0.76 (0.57-0.89) | |
| F1 E | -3801 | -1423 | - | - | - | 0.58 (0.32-0.69) | 0.89 (0.75-0.96) | - | - | - | 0.76 (0.68-0.83) | |
| E1 P | -3670 | -1488 | - | - | - | 0.57 (0.45-0.68) | 0.89 (0.77-0.95) | - | - | - | 0.77 (0.59-0.88) | |
| E1 T | -186 | -1530 | - | - | - | 0.21 (0.06-0.40) | 0.91 (0.80-0.96) | - | - | - | 0.87 (0.75-0.95) | |
| E1 S | -5542 | -1665 | - | - | - | 0.69 (0.52-0.81) | 0.87 (0.76-0.94) | - | - | - | 0.56 (0.49-0.61) | |
| E1 F | -6220 | -1545 | - | - | - | 0.72 (0.51-0.85) | 0.89 (0.82-0.93) | - | - | - | 0.72 (0.52-0.88) | |
| E1 E | -5036 | -1640 | - | - | - | 0.66 (0.53-0.77) | 0.90 (0.78-0.96) | - | - | - | 0.61 (0.53-0.69) | |
| M M1 | -2218 | -1491 | -1262 | -1112 | -879 | 0.45 (0.19-0.58) | 0.90 (0.79-0.97) | 0.86 (0.78-0.91) | 0.89 (0.79-0.93) | 0.88 (0.78-0.92) | 0.83 (0.75-0.90) | |
| M E1 | -3746 | -1585 | -1381 | -1179 | -968 | 0.58 (0.47-0.67) | 0.88 (0.76-0.94) | 0.87 (0.77-0.93) | 0.86 (0.74-0.91) | 0.88 (0.76-0.92) | 0.67 (0.60-0.75) | |
| M F1 | -1456 | -1370 | -1197 | -1002 | -832 | 0.37 (0.13-0.52) | 0.91 (0.81-0.96) | 0.90 (0.80-0.94) | 0.90 (0.78-0.94) | 0.90 (0.81-0.95) | 0.86 (0.76-0.93) | |
| M S1 | -1519 | -1512 | -1309 | -1123 | -885 | 0.38 (0.13-0.56) | 0.91 (0.81-0.96) | 0.89 (0.78-0.93) | 0.90 (0.81-0.94) | 0.90 (0.81-0.93) | 0.86 (0.76-0.93) | |
| M T1 | -1904 | -1469 | -1262 | -1037 | -864 | 0.42 (0.14-0.63) | 0.91 (0.82-0.96) | 0.89 (0.78-0.93) | 0.88 (0.78-0.93) | 0.88 (0.77-0.92) | 0.83 (0.69-0.93) | |
| M P1 | -1479 | -1430 | -1250 | -1056 | -865 | 0.37 (0.13-0.53) | 0.91 (0.82-0.96) | 0.90 (0.79-0.95) | 0.88 (0.78-0.92) | 0.89 (0.80-0.93) | 0.86 (0.77-0.92) | |
| E M1 | -3834 | -1486 | -1312 | -1134 | -912 | 0.58 (0.32-0.69) | 0.90 (0.79-0.96) | 0.91 (0.83-0.95) | 0.89 (0.79-0.93) | 0.86 (0.76-0.90) | 0.77 (0.67-0.86) | |
| F M1 | -5705 | -1427 | -1247 | -1039 | -837 | 0.70 (0.52-0.81) | 0.88 (0.77-0.94) | 0.81 (0.70-0.88) | 0.82 (0.71-0.88) | 0.80 (0.72-0.86) | 0.82 (0.67-0.89) | |
| S M1 | -3063 | -1534 | -1306 | -1146 | -919 | 0.52 (0.25-0.77) | 0.90 (0.82-0.94) | 0.88 (0.77-0.93) | 0.89 (0.78-0.94) | 0.89 (0.78-0.95) | 0.77 (0.58-0.90) | |
| T M1 | 691 | -1452 | -1272 | -1073 | -877 | 0.08 (0.00-0.29) | 0.91 (0.79-0.96) | 0.90 (0.78-0.95) | 0.88 (0.77-0.92) | 0.90 (0.78-0.94) | 0.93 (0.83-0.98) | |
| P M1 | -3502 | -1416 | -1207 | -1022 | -835 | 0.56 (0.44-0.64) | 0.87 (0.72-0.94) | 0.84 (0.72-0.90) | 0.86 (0.73-0.91) | 0.86 (0.73-0.92) | 0.76 (0.57-0.86) | |
| P1 P T | -3463 | -1230 | - | - | - | 0.59 (0.52-0.66) | 0.87 (0.77-0.93) | - | - | - | 0.75 (0.60-0.83) | |
| P1 P S | -7128 | -1368 | - | - | - | 0.78 (0.62-0.89) | 0.85 (0.72-0.91) | - | - | - | 0.50 (0.29-0.59) | |
| P1 P F | -7167 | -1251 | - | - | - | 0.78 (0.62-0.87) | 0.89 (0.81-0.93) | - | - | - | 0.59 (0.34-0.76) | |
| P1 P E | -6820 | -1321 | - | - | - | 0.77 (0.65-0.84) | 0.84 (0.68-0.92) | - | - | - | 0.53 (0.39-0.65) | |
| P1 T S | -4773 | -1399 | - | - | - | 0.67 (0.52-0.82) | 0.81 (0.68-0.88) | - | - | - | 0.58 (0.40-0.69) | |
| P1 T F | -5841 | -1296 | - | - | - | 0.73 (0.53-0.86) | 0.88 (0.83-0.91) | - | - | - | 0.74 (0.54-0.87) | |
| P1 T E | -8120 | -1445 | - | - | - | 0.82 (0.73-0.86) | 0.87 (0.77-0.91) | - | - | - | 0.34 (0.19-0.50) | |
| P1 S F | -8338 | -1396 | - | - | - | 0.82 (0.70-0.87) | 0.89 (0.82-0.93) | - | - | - | 0.50 (0.32-0.61) | |
| P1 S E | -3984 | -1316 | - | - | - | 0.62 (0.33-0.80) | 0.92 (0.82-0.97) | - | - | - | 0.72 (0.59-0.85) | |
| P1 F E | -7147 | -1323 | - | - | - | 0.78 (0.68-0.86) | 0.87 (0.74-0.92) | - | - | - | 0.59 (0.47-0.71) | |
| T1 P T | -3487 | -1223 | - | - | - | 0.59 (0.51-0.67) | 0.89 (0.78-0.94) | - | - | - | 0.77 (0.63-0.85) | |
| T1 P S | -7236 | -1393 | - | - | - | 0.78 (0.63-0.90) | 0.82 (0.67-0.90) | - | - | - | 0.34 (0.14-0.42) | |
| T1 P F | -7163 | -1247 | - | - | - | 0.78 (0.61-0.88) | 0.90 (0.82-0.94) | - | - | - | 0.61 (0.37-0.76) | |
| T1 P E | -6899 | -1284 | - | - | - | 0.77 (0.65-0.84) | 0.85 (0.71-0.92) | - | - | - | 0.60 (0.49-0.69) | |
| T1 T S | -4791 | -1406 | - | - | - | 0.67 (0.51-0.83) | 0.79 (0.64-0.87) | - | - | - | 0.49 (0.33-0.60) | |
| T1 T F | -5847 | -1309 | - | - | - | 0.73 (0.53-0.86) | 0.88 (0.84-0.91) | - | - | - | 0.73 (0.56-0.85) | |
| T1 T E | -8640 | -1420 | - | - | - | 0.83 (0.76-0.87) | 0.87 (0.76-0.91) | - | - | - | 0.32 (0.18-0.45) | |
| T1 S F | -8389 | -1386 | - | - | - | 0.82 (0.70-0.87) | 0.90 (0.83-0.94) | - | - | - | 0.53 (0.38-0.64) | |
| T1 S E | -4032 | -1304 | - | - | - | 0.62 (0.33-0.82) | 0.92 (0.84-0.97) | - | - | - | 0.74 (0.57-0.88) | |
| T1 F E | -7356 | -1312 | - | - | - | 0.79 (0.69-0.86) | 0.89 (0.78-0.94) | - | - | - | 0.63 (0.56-0.70) | |
| S1 P T | -3470 | -1289 | - | - | - | 0.59 (0.51-0.66) | 0.89 (0.78-0.94) | - | - | - | 0.78 (0.64-0.86) | |
| S1 P S | -7337 | -1423 | - | - | - | 0.79 (0.63-0.89) | 0.85 (0.70-0.91) | - | - | - | 0.51 (0.27-0.58) | |
| S1 P F | -7119 | -1307 | - | - | - | 0.78 (0.61-0.88) | 0.90 (0.82-0.94) | - | - | - | 0.62 (0.38-0.77) | |
| S1 P E | -6828 | -1380 | - | - | - | 0.77 (0.65-0.84) | 0.84 (0.69-0.92) | - | - | - | 0.55 (0.41-0.65) | |
| S1 T S | -4854 | -1418 | - | - | - | 0.67 (0.52-0.82) | 0.86 (0.73-0.92) | - | - | - | 0.67 (0.52-0.77) | |
| S1 T F | -5761 | -1356 | - | - | - | 0.72 (0.52-0.86) | 0.89 (0.84-0.92) | - | - | - | 0.76 (0.58-0.88) | |
| S1 T E | -8364 | -1491 | - | - | - | 0.82 (0.75-0.87) | 0.87 (0.76-0.91) | - | - | - | 0.33 (0.19-0.48) | |
| S1 S F | -8458 | -1437 | - | - | - | 0.83 (0.71-0.88) | 0.90 (0.83-0.94) | - | - | - | 0.54 (0.38-0.64) | |
| S1 S E | -3940 | -1363 | - | - | - | 0.62 (0.33-0.81) | 0.92 (0.81-0.97) | - | - | - | 0.71 (0.57-0.83) | |
| S1 F E | -7196 | -1378 | - | - | - | 0.78 (0.68-0.87) | 0.88 (0.76-0.93) | - | - | - | 0.61 (0.51-0.71) | |
| F1 P T | -3478 | -1166 | - | - | - | 0.59 (0.51-0.67) | 0.89 (0.79-0.94) | - | - | - | 0.78 (0.64-0.86) | |
| F1 P S | -7063 | -1326 | - | - | - | 0.78 (0.62-0.89) | 0.84 (0.70-0.90) | - | - | - | 0.46 (0.23-0.57) | |
| F1 P F | -7040 | -1179 | - | - | - | 0.78 (0.59-0.87) | 0.90 (0.81-0.94) | - | - | - | 0.62 (0.37-0.79) | |
| F1 P E | -6768 | -1272 | - | - | - | 0.77 (0.65-0.84) | 0.83 (0.67-0.92) | - | - | - | 0.51 (0.36-0.64) | |
| F1 T S | -4757 | -1318 | - | - | - | 0.67 (0.51-0.82) | 0.85 (0.73-0.91) | - | - | - | 0.66 (0.49-0.76) | |
| F1 T F | -5715 | -1228 | - | - | - | 0.72 (0.51-0.86) | 0.90 (0.84-0.94) | - | - | - | 0.79 (0.61-0.90) | |
| F1 T E | -8082 | -1392 | - | - | - | 0.81 (0.72-0.86) | 0.87 (0.77-0.91) | - | - | - | 0.35 (0.21-0.51) | |
| F1 S F | -8340 | -1334 | - | - | - | 0.82 (0.70-0.87) | 0.90 (0.83-0.93) | - | - | - | 0.52 (0.35-0.63) | |
| F1 S E | -3929 | -1256 | - | - | - | 0.62 (0.33-0.80) | 0.92 (0.82-0.96) | - | - | - | 0.72 (0.59-0.85) | |
| F1 F E | -7122 | -1260 | - | - | - | 0.78 (0.67-0.86) | 0.88 (0.76-0.93) | - | - | - | 0.63 (0.53-0.72) | |
| E1 P T | -3812 | -1293 | - | - | - | 0.61 (0.52-0.69) | 0.90 (0.79-0.95) | - | - | - | 0.77 (0.60-0.87) | |
| E1 P S | -8035 | -1419 | - | - | - | 0.81 (0.68-0.89) | 0.87 (0.72-0.94) | - | - | - | 0.43 (0.35-0.51) | |
| E1 P F | -7342 | -1323 | - | - | - | 0.79 (0.61-0.89) | 0.91 (0.84-0.94) | - | - | - | 0.62 (0.38-0.79) | |
| E1 P E | -7193 | -1389 | - | - | - | 0.78 (0.69-0.85) | 0.87 (0.75-0.93) | - | - | - | 0.54 (0.40-0.64) | |
| E1 T S | -5889 | -1446 | - | - | - | 0.73 (0.59-0.82) | 0.88 (0.74-0.94) | - | - | - | 0.60 (0.55-0.65) | |
| E1 T F | -6011 | -1381 | - | - | - | 0.73 (0.51-0.88) | 0.90 (0.84-0.93) | - | - | - | 0.70 (0.51-0.87) | |
| E1 T E | -8367 | -1452 | - | - | - | 0.82 (0.75-0.86) | 0.87 (0.77-0.92) | - | - | - | 0.36 (0.22-0.51) | |
| E1 S F | -9944 | -1524 | - | - | - | 0.87 (0.80-0.92) | 0.91 (0.85-0.94) | - | - | - | 0.33 (0.23-0.41) | |
| E1 S E | -5830 | -1468 | - | - | - | 0.73 (0.56-0.82) | 0.91 (0.79-0.96) | - | - | - | 0.51 (0.43-0.58) | |
| E1 F E | -7879 | -1451 | - | - | - | 0.81 (0.67-0.89) | 0.91 (0.82-0.95) | - | - | - | 0.51 (0.38-0.66) | |
| M E M1 | -3524 | -1223 | -1032 | -816 | -646 | 0.59 (0.35-0.69) | 0.90 (0.81-0.96) | 0.87 (0.77-0.92) | 0.88 (0.79-0.92) | 0.88 (0.80-0.92) | 0.76 (0.67-0.85) | |
| M F M1 | -6872 | -1336 | -1173 | -983 | -773 | 0.77 (0.68-0.85) | 0.88 (0.77-0.93) | 0.87 (0.78-0.91) | 0.87 (0.80-0.91) | 0.86 (0.79-0.90) | 0.67 (0.57-0.75) | |
| M S M1 | -2908 | -1328 | -1139 | -917 | -732 | 0.54 (0.27-0.78) | 0.91 (0.82-0.95) | 0.89 (0.78-0.94) | 0.88 (0.78-0.91) | 0.89 (0.77-0.95) | 0.75 (0.57-0.89) | |
| T M M1 | -6014 | -1400 | -1231 | -1035 | -824 | 0.73 (0.64-0.78) | 0.84 (0.70-0.92) | 0.84 (0.71-0.91) | 0.85 (0.71-0.91) | 0.84 (0.71-0.91) | 0.47 (0.35-0.60) | |
| P M M1 | -6150 | -1314 | -1148 | -953 | -700 | 0.74 (0.61-0.81) | 0.84 (0.70-0.92) | 0.84 (0.71-0.91) | 0.85 (0.73-0.91) | 0.84 (0.73-0.90) | 0.62 (0.51-0.71) | |
| M E E1 | -4758 | -1357 | -1141 | -955 | -719 | 0.67 (0.53-0.78) | 0.90 (0.80-0.95) | 0.90 (0.80-0.94) | 0.89 (0.78-0.93) | 0.88 (0.77-0.92) | 0.61 (0.53-0.68) | |
| M E F1 | -3477 | -1161 | -992 | -767 | -575 | 0.59 (0.34-0.69) | 0.90 (0.79-0.95) | 0.89 (0.79-0.93) | 0.89 (0.80-0.94) | 0.90 (0.81-0.94) | 0.75 (0.67-0.83) | |
| M E S1 | -3532 | -1297 | -1094 | -879 | -655 | 0.59 (0.34-0.71) | 0.91 (0.82-0.96) | 0.89 (0.79-0.93) | 0.89 (0.80-0.93) | 0.89 (0.79-0.93) | 0.77 (0.67-0.86) | |
| M E T1 | -3749 | -1247 | -1043 | -831 | -640 | 0.61 (0.34-0.74) | 0.91 (0.83-0.96) | 0.88 (0.79-0.92) | 0.89 (0.81-0.93) | 0.88 (0.78-0.92) | 0.76 (0.63-0.87) | |
| M E P1 | -3525 | -1217 | -1053 | -848 | -672 | 0.59 (0.35-0.70) | 0.91 (0.81-0.96) | 0.89 (0.78-0.93) | 0.89 (0.79-0.93) | 0.89 (0.79-0.93) | 0.77 (0.69-0.85) | |
| M F E1 | -7787 | -1432 | -1212 | -1042 | -842 | 0.80 (0.68-0.88) | 0.89 (0.81-0.93) | 0.87 (0.81-0.91) | 0.86 (0.80-0.90) | 0.85 (0.78-0.90) | 0.49 (0.35-0.64) | |
| M F F1 | -6696 | -1201 | -1039 | -876 | -679 | 0.76 (0.68-0.83) | 0.89 (0.80-0.94) | 0.86 (0.78-0.91) | 0.85 (0.77-0.89) | 0.84 (0.77-0.90) | 0.73 (0.67-0.78) | |
| M F S1 | -6779 | -1338 | -1121 | -956 | -789 | 0.77 (0.69-0.83) | 0.90 (0.81-0.94) | 0.85 (0.78-0.89) | 0.84 (0.75-0.89) | 0.84 (0.77-0.90) | 0.74 (0.68-0.81) | |
| M F T1 | -7065 | -1304 | -1113 | -901 | -706 | 0.78 (0.69-0.83) | 0.89 (0.83-0.94) | 0.86 (0.79-0.91) | 0.84 (0.76-0.90) | 0.85 (0.76-0.90) | 0.69 (0.60-0.75) | |
| M F P1 | -6755 | -1258 | -1106 | -924 | -735 | 0.77 (0.68-0.83) | 0.89 (0.81-0.94) | 0.85 (0.78-0.90) | 0.84 (0.76-0.90) | 0.84 (0.75-0.89) | 0.73 (0.67-0.79) | |
| M S E1 | -5406 | -1426 | -1210 | -1014 | -851 | 0.70 (0.53-0.81) | 0.89 (0.77-0.95) | 0.88 (0.75-0.94) | 0.88 (0.76-0.95) | 0.87 (0.73-0.93) | 0.56 (0.49-0.62) | |
| M S F1 | -2674 | -1225 | -1017 | -850 | -664 | 0.53 (0.25-0.78) | 0.91 (0.82-0.95) | 0.85 (0.74-0.91) | 0.88 (0.77-0.94) | 0.90 (0.79-0.95) | 0.77 (0.59-0.90) | |
| M S S1 | -2765 | -1340 | -1148 | -937 | -762 | 0.53 (0.26-0.78) | 0.92 (0.83-0.95) | 0.89 (0.77-0.95) | 0.88 (0.75-0.93) | 0.89 (0.77-0.95) | 0.79 (0.62-0.91) | |
| M S T1 | -2738 | -1299 | -1082 | -893 | -702 | 0.53 (0.25-0.79) | 0.92 (0.81-0.97) | 0.89 (0.77-0.94) | 0.88 (0.76-0.94) | 0.90 (0.77-0.95) | 0.75 (0.57-0.89) | |
| M S P1 | -2713 | -1289 | -1103 | -916 | -731 | 0.53 (0.26-0.78) | 0.91 (0.81-0.95) | 0.89 (0.78-0.95) | 0.88 (0.78-0.94) | 0.90 (0.77-0.95) | 0.75 (0.58-0.88) | |
| T M E1 | -4525 | -1392 | -1158 | -946 | -746 | 0.66 (0.59-0.71) | 0.88 (0.76-0.93) | 0.87 (0.75-0.93) | 0.88 (0.76-0.93) | 0.86 (0.75-0.92) | 0.58 (0.48-0.69) | |
| T M F1 | -3795 | -1250 | -1090 | -892 | -709 | 0.61 (0.54-0.65) | 0.87 (0.75-0.93) | 0.87 (0.75-0.93) | 0.88 (0.75-0.93) | 0.87 (0.75-0.94) | 0.62 (0.55-0.67) | |
| T M S1 | -4079 | -1392 | -1213 | -1010 | -779 | 0.63 (0.55-0.68) | 0.86 (0.75-0.91) | 0.87 (0.74-0.92) | 0.87 (0.74-0.92) | 0.86 (0.74-0.92) | 0.59 (0.50-0.65) | |
| T M T1 | -4709 | -1357 | -1157 | -957 | -735 | 0.67 (0.56-0.75) | 0.86 (0.75-0.91) | 0.85 (0.74-0.89) | 0.85 (0.74-0.89) | 0.85 (0.73-0.91) | 0.56 (0.43-0.64) | |
| T M P1 | -3885 | -1309 | -1152 | -887 | -753 | 0.62 (0.54-0.66) | 0.86 (0.75-0.92) | 0.87 (0.75-0.93) | 0.86 (0.75-0.91) | 0.87 (0.76-0.93) | 0.61 (0.53-0.66) | |
| P M E1 | -6124 | -1364 | -1145 | -949 | -736 | 0.74 (0.66-0.80) | 0.85 (0.72-0.91) | 0.85 (0.72-0.90) | 0.85 (0.75-0.91) | 0.84 (0.73-0.89) | 0.55 (0.44-0.67) | |
| P M F1 | -5419 | -1213 | -1018 | -857 | -646 | 0.71 (0.60-0.78) | 0.82 (0.68-0.90) | 0.81 (0.69-0.87) | 0.83 (0.71-0.90) | 0.81 (0.70-0.87) | 0.60 (0.51-0.65) | |
| P M S1 | -5470 | -1357 | -1157 | -934 | -717 | 0.71 (0.60-0.79) | 0.83 (0.70-0.90) | 0.84 (0.71-0.89) | 0.85 (0.72-0.90) | 0.84 (0.72-0.89) | 0.62 (0.52-0.67) | |
| P M T1 | -5612 | -1294 | -1101 | -889 | -682 | 0.71 (0.60-0.81) | 0.84 (0.71-0.90) | 0.84 (0.72-0.90) | 0.83 (0.70-0.88) | 0.83 (0.70-0.89) | 0.63 (0.50-0.68) | |
| P M P1 | -5446 | -1268 | -1056 | -914 | -713 | 0.71 (0.60-0.78) | 0.84 (0.71-0.90) | 0.82 (0.70-0.88) | 0.83 (0.72-0.89) | 0.83 (0.71-0.88) | 0.63 (0.53-0.68) | |
| P1 T1 P | -3135 | -1134 | - | - | - | 0.56 (0.47-0.64) | 0.87 (0.74-0.94) | - | - | - | 0.75 (0.58-0.84) | |
| P1 S1 P | -3115 | -1201 | - | - | - | 0.56 (0.47-0.63) | 0.88 (0.74-0.94) | - | - | - | 0.77 (0.59-0.85) | |
| P1 F1 P | -3127 | -1079 | - | - | - | 0.56 (0.46-0.64) | 0.87 (0.73-0.93) | - | - | - | 0.74 (0.56-0.83) | |
| P1 E1 P | -3319 | -1215 | - | - | - | 0.58 (0.47-0.68) | 0.89 (0.76-0.95) | - | - | - | 0.76 (0.57-0.86) | |
| T1 S1 P | -3088 | -1402 | - | - | - | 0.56 (0.46-0.64) | 0.88 (0.74-0.95) | - | - | - | 0.78 (0.60-0.86) | |
| T1 F1 P | -3133 | -1067 | - | - | - | 0.56 (0.47-0.64) | 0.88 (0.73-0.95) | - | - | - | 0.76 (0.58-0.85) | |
| T1 E1 P | -3282 | -1215 | - | - | - | 0.57 (0.46-0.68) | 0.89 (0.76-0.95) | - | - | - | 0.75 (0.56-0.85) | |
| S1 F1 P | -3106 | -1143 | - | - | - | 0.56 (0.46-0.64) | 0.88 (0.73-0.95) | - | - | - | 0.76 (0.58-0.86) | |
| S1 E1 P | -3279 | -1257 | - | - | - | 0.57 (0.46-0.68) | 0.89 (0.76-0.95) | - | - | - | 0.75 (0.57-0.86) | |
| F1 E1 P | -3326 | -1170 | - | - | - | 0.58 (0.45-0.69) | 0.89 (0.76-0.95) | - | - | - | 0.74 (0.54-0.86) | |
| P1 T1 T | 952 | -1159 | - | - | - | 0.11 (0.05-0.29) | 0.91 (0.81-0.96) | - | - | - | 0.93 (0.85-0.98) | |
| P1 S1 T | 1067 | -1245 | - | - | - | 0.09 (0.04-0.28) | 0.91 (0.80-0.96) | - | - | - | 0.93 (0.84-0.98) | |
| P1 F1 T | 1096 | -1098 | - | - | - | 0.09 (0.03-0.28) | 0.91 (0.80-0.96) | - | - | - | 0.93 (0.83-0.98) | |
| P1 E1 T | 77 | -1246 | - | - | - | 0.23 (0.10-0.41) | 0.91 (0.81-0.96) | - | - | - | 0.88 (0.75-0.94) | |
| T1 S1 T | 984 | -1206 | - | - | - | 0.10 (0.04-0.28) | 0.91 (0.80-0.96) | - | - | - | 0.93 (0.84-0.97) | |
| T1 F1 T | 942 | -1102 | - | - | - | 0.11 (0.05-0.29) | 0.91 (0.79-0.96) | - | - | - | 0.93 (0.84-0.97) | |
| T1 E1 T | -368 | -1247 | - | - | - | 0.29 (0.16-0.44) | 0.90 (0.80-0.95) | - | - | - | 0.84 (0.73-0.92) | |
| S1 F1 T | 1117 | -1164 | - | - | - | 0.08 (0.03-0.29) | 0.91 (0.79-0.96) | - | - | - | 0.93 (0.84-0.98) | |
| S1 E1 T | -186 | -1296 | - | - | - | 0.27 (0.12-0.45) | 0.91 (0.80-0.95) | - | - | - | 0.86 (0.75-0.93) | |
| F1 E1 T | 181 | -1206 | - | - | - | 0.22 (0.06-0.41) | 0.91 (0.80-0.96) | - | - | - | 0.87 (0.75-0.95) | |
| P1 T1 S | -2640 | -1251 | - | - | - | 0.52 (0.26-0.78) | 0.90 (0.81-0.95) | - | - | - | 0.76 (0.59-0.89) | |
| P1 S1 S | -2739 | -1333 | - | - | - | 0.53 (0.27-0.77) | 0.90 (0.81-0.96) | - | - | - | 0.77 (0.63-0.88) | |
| P1 F1 S | -2616 | -1197 | - | - | - | 0.52 (0.26-0.77) | 0.90 (0.80-0.94) | - | - | - | 0.76 (0.58-0.88) | |
| P1 E1 S | -5141 | -1387 | - | - | - | 0.69 (0.52-0.81) | 0.87 (0.76-0.95) | - | - | - | 0.56 (0.50-0.62) | |
| T1 S1 S | -2932 | -1291 | - | - | - | 0.55 (0.27-0.79) | 0.90 (0.81-0.95) | - | - | - | 0.77 (0.59-0.90) | |
| T1 F1 S | -2590 | -1187 | - | - | - | 0.52 (0.25-0.78) | 0.90 (0.81-0.95) | - | - | - | 0.76 (0.57-0.89) | |
| T1 E1 S | -5438 | -1377 | - | - | - | 0.71 (0.54-0.83) | 0.88 (0.76-0.94) | - | - | - | 0.58 (0.52-0.65) | |
| S1 F1 S | -2721 | -1246 | - | - | - | 0.53 (0.26-0.77) | 0.90 (0.81-0.95) | - | - | - | 0.76 (0.59-0.89) | |
| S1 E1 S | -5208 | -1425 | - | - | - | 0.69 (0.53-0.81) | 0.87 (0.76-0.94) | - | - | - | 0.57 (0.50-0.62) | |
| F1 E1 S | -5161 | -1344 | - | - | - | 0.69 (0.53-0.81) | 0.87 (0.76-0.94) | - | - | - | 0.56 (0.49-0.62) | |
| P1 T1 F | -5493 | -1164 | - | - | - | 0.71 (0.54-0.81) | 0.86 (0.76-0.92) | - | - | - | 0.76 (0.66-0.83) | |
| P1 S1 F | -5436 | -1215 | - | - | - | 0.71 (0.53-0.81) | 0.87 (0.74-0.91) | - | - | - | 0.76 (0.60-0.87) | |
| P1 F1 F | -5396 | -1099 | - | - | - | 0.70 (0.53-0.81) | 0.86 (0.73-0.91) | - | - | - | 0.75 (0.56-0.88) | |
| P1 E1 F | -5972 | -1267 | - | - | - | 0.73 (0.53-0.85) | 0.89 (0.81-0.93) | - | - | - | 0.71 (0.51-0.86) | |
| T1 S1 F | -5431 | -1211 | - | - | - | 0.71 (0.53-0.81) | 0.87 (0.74-0.92) | - | - | - | 0.77 (0.60-0.88) | |
| T1 F1 F | -5408 | -1108 | - | - | - | 0.70 (0.53-0.81) | 0.86 (0.74-0.91) | - | - | - | 0.75 (0.58-0.85) | |
| T1 E1 F | -6037 | -1268 | - | - | - | 0.74 (0.53-0.86) | 0.89 (0.82-0.93) | - | - | - | 0.70 (0.51-0.84) | |
| S1 F1 F | -5337 | -1157 | - | - | - | 0.70 (0.52-0.81) | 0.88 (0.75-0.93) | - | - | - | 0.79 (0.63-0.89) | |
| S1 E1 F | -5938 | -1310 | - | - | - | 0.73 (0.52-0.86) | 0.89 (0.82-0.93) | - | - | - | 0.72 (0.54-0.89) | |
| F1 E1 F | -5813 | -1220 | - | - | - | 0.72 (0.51-0.86) | 0.90 (0.82-0.94) | - | - | - | 0.74 (0.54-0.90) | |
| P1 T1 E | -3685 | -1203 | - | - | - | 0.60 (0.33-0.73) | 0.89 (0.76-0.95) | - | - | - | 0.75 (0.66-0.83) | |
| P1 S1 E | -3478 | -1263 | - | - | - | 0.59 (0.32-0.70) | 0.90 (0.77-0.96) | - | - | - | 0.75 (0.68-0.83) | |
| P1 F1 E | -3433 | -1144 | - | - | - | 0.58 (0.32-0.70) | 0.89 (0.76-0.96) | - | - | - | 0.76 (0.69-0.83) | |
| P1 E1 E | -4642 | -1355 | - | - | - | 0.66 (0.53-0.77) | 0.91 (0.79-0.96) | - | - | - | 0.62 (0.54-0.69) | |
| T1 S1 E | -3735 | -1247 | - | - | - | 0.60 (0.33-0.73) | 0.90 (0.80-0.95) | - | - | - | 0.76 (0.64-0.87) | |
| T1 F1 E | -3642 | -1138 | - | - | - | 0.60 (0.32-0.73) | 0.91 (0.81-0.96) | - | - | - | 0.76 (0.63-0.87) | |
| T1 E1 E | -5309 | -1360 | - | - | - | 0.70 (0.56-0.79) | 0.90 (0.79-0.95) | - | - | - | 0.57 (0.49-0.64) | |
| S1 F1 E | -3435 | -1212 | - | - | - | 0.58 (0.32-0.69) | 0.89 (0.73-0.96) | - | - | - | 0.75 (0.68-0.81) | |
| S1 E1 E | -4986 | -1409 | - | - | - | 0.68 (0.56-0.78) | 0.90 (0.79-0.95) | - | - | - | 0.58 (0.50-0.66) | |
| F E M1 | -7195 | -1281 | -1119 | -938 | -766 | 0.78 (0.69-0.86) | 0.90 (0.83-0.95) | 0.89 (0.82-0.93) | 0.88 (0.80-0.92) | 0.87 (0.79-0.91) | 0.71 (0.63-0.76) | |
| S E M1 | -3938 | -1318 | -1118 | -946 | -650 | 0.62 (0.33-0.80) | 0.92 (0.82-0.97) | 0.90 (0.79-0.94) | 0.90 (0.80-0.95) | 0.90 (0.78-0.95) | 0.74 (0.59-0.86) | |
| S F M1 | -8332 | -1399 | -1189 | -1026 | -788 | 0.82 (0.70-0.87) | 0.89 (0.81-0.93) | 0.89 (0.80-0.94) | 0.89 (0.80-0.92) | 0.89 (0.82-0.92) | 0.52 (0.35-0.60) | |
| T E M1 | -8158 | -1455 | -1249 | -1046 | -836 | 0.82 (0.74-0.86) | 0.87 (0.76-0.90) | 0.87 (0.77-0.90) | 0.87 (0.77-0.91) | 0.87 (0.77-0.91) | 0.32 (0.19-0.49) | |
| T F M1 | -5738 | -1294 | -1133 | -905 | -705 | 0.72 (0.52-0.86) | 0.90 (0.84-0.94) | 0.82 (0.71-0.88) | 0.83 (0.70-0.89) | 0.83 (0.72-0.89) | 0.79 (0.61-0.89) | |
| T S M1 | -4912 | -1374 | -1208 | -985 | -777 | 0.68 (0.52-0.82) | 0.86 (0.73-0.92) | 0.79 (0.66-0.85) | 0.83 (0.68-0.92) | 0.82 (0.67-0.92) | 0.68 (0.53-0.77) | |
| P E M1 | -6808 | -1306 | -1135 | -951 | -741 | 0.77 (0.65-0.84) | 0.84 (0.69-0.92) | 0.84 (0.71-0.90) | 0.85 (0.73-0.91) | 0.84 (0.72-0.90) | 0.57 (0.46-0.67) | |
| P F M1 | -7064 | -1240 | -1051 | -886 | -691 | 0.78 (0.59-0.87) | 0.90 (0.82-0.94) | 0.86 (0.77-0.91) | 0.88 (0.83-0.91) | 0.88 (0.83-0.92) | 0.66 (0.43-0.82) | |
| P S M1 | -7157 | -1386 | -1141 | -951 | -776 | 0.78 (0.62-0.89) | 0.84 (0.71-0.90) | 0.85 (0.72-0.91) | 0.85 (0.70-0.94) | 0.84 (0.69-0.93) | 0.46 (0.25-0.54) | |
| P T M1 | -3503 | -1241 | -1048 | -875 | -697 | 0.59 (0.51-0.67) | 0.89 (0.79-0.94) | 0.86 (0.77-0.90) | 0.83 (0.75-0.87) | 0.82 (0.73-0.85) | 0.78 (0.64-0.86) | |
| M F1 E1 | -3362 | -1257 | -1036 | -809 | -651 | 0.58 (0.48-0.67) | 0.89 (0.77-0.94) | 0.87 (0.74-0.92) | 0.87 (0.75-0.92) | 0.86 (0.75-0.91) | 0.68 (0.62-0.76) | |
| M S1 E1 | -4220 | -1374 | -1128 | -923 | -738 | 0.64 (0.51-0.72) | 0.87 (0.75-0.92) | 0.87 (0.75-0.92) | 0.87 (0.76-0.92) | 0.84 (0.74-0.89) | 0.62 (0.56-0.71) | |
| M S1 F1 | -1104 | -1179 | -986 | -783 | -561 | 0.38 (0.13-0.56) | 0.92 (0.81-0.96) | 0.89 (0.76-0.94) | 0.87 (0.78-0.92) | 0.83 (0.72-0.88) | 0.86 (0.75-0.92) | |
| M T1 E1 | -5016 | -1324 | -1096 | -912 | -698 | 0.68 (0.54-0.76) | 0.86 (0.76-0.91) | 0.85 (0.73-0.89) | 0.84 (0.74-0.89) | 0.83 (0.73-0.87) | 0.63 (0.58-0.70) | |
| M T1 F1 | -1526 | -1131 | -924 | -727 | -490 | 0.42 (0.15-0.64) | 0.91 (0.82-0.96) | 0.87 (0.76-0.92) | 0.88 (0.77-0.92) | 0.87 (0.77-0.91) | 0.84 (0.71-0.93) | |
| M T1 S1 | -1672 | -1233 | -1010 | -854 | -621 | 0.43 (0.16-0.63) | 0.91 (0.81-0.95) | 0.85 (0.73-0.90) | 0.86 (0.79-0.90) | 0.82 (0.70-0.87) | 0.85 (0.73-0.94) | |
| M P1 E1 | -3440 | -1304 | -1076 | -853 | -737 | 0.58 (0.48-0.68) | 0.88 (0.76-0.93) | 0.87 (0.76-0.92) | 0.85 (0.75-0.90) | 0.87 (0.76-0.91) | 0.66 (0.61-0.74) | |
| M P1 F1 | -1068 | -1096 | -881 | -730 | -521 | 0.37 (0.13-0.53) | 0.92 (0.82-0.96) | 0.88 (0.79-0.93) | 0.90 (0.80-0.94) | 0.89 (0.80-0.93) | 0.86 (0.76-0.92) | |
| M P1 S1 | -1123 | -1237 | -1041 | -833 | -625 | 0.38 (0.13-0.57) | 0.92 (0.82-0.97) | 0.89 (0.78-0.93) | 0.87 (0.77-0.91) | 0.87 (0.76-0.92) | 0.85 (0.74-0.92) | |
| M P1 T1 | -1505 | -1193 | -968 | -767 | -546 | 0.42 (0.15-0.63) | 0.91 (0.82-0.96) | 0.87 (0.77-0.91) | 0.88 (0.77-0.92) | 0.84 (0.72-0.90) | 0.83 (0.70-0.92) | |
| E M1 E1 | -4801 | -1362 | -1147 | -974 | -771 | 0.67 (0.54-0.78) | 0.90 (0.79-0.95) | 0.89 (0.79-0.94) | 0.89 (0.78-0.94) | 0.89 (0.79-0.94) | 0.61 (0.54-0.69) | |
| E M1 F1 | -3423 | -1160 | -978 | -775 | -534 | 0.58 (0.32-0.69) | 0.88 (0.73-0.95) | 0.89 (0.78-0.93) | 0.89 (0.78-0.93) | 0.90 (0.81-0.93) | 0.74 (0.68-0.80) | |
| E M1 S1 | -3470 | -1293 | -1099 | -874 | -691 | 0.59 (0.32-0.70) | 0.90 (0.78-0.95) | 0.88 (0.77-0.93) | 0.88 (0.77-0.92) | 0.88 (0.79-0.92) | 0.75 (0.67-0.84) | |
| E T1 M1 | -3700 | -1258 | -1026 | -783 | -592 | 0.60 (0.32-0.73) | 0.91 (0.80-0.96) | 0.88 (0.79-0.92) | 0.88 (0.80-0.92) | 0.89 (0.80-0.92) | 0.77 (0.65-0.87) | |
| E P1 M1 | -3468 | -1209 | -1034 | -801 | -647 | 0.59 (0.33-0.70) | 0.90 (0.79-0.96) | 0.89 (0.79-0.93) | 0.87 (0.77-0.92) | 0.88 (0.77-0.91) | 0.75 (0.66-0.84) | |
| F M1 E1 | -5879 | -1264 | -1046 | -848 | -638 | 0.73 (0.52-0.86) | 0.90 (0.82-0.94) | 0.83 (0.71-0.89) | 0.81 (0.69-0.88) | 0.82 (0.70-0.89) | 0.75 (0.54-0.91) | |
| F M1 F1 | -5298 | -1101 | -890 | -718 | -477 | 0.70 (0.52-0.81) | 0.88 (0.74-0.93) | 0.79 (0.68-0.86) | 0.81 (0.72-0.88) | 0.78 (0.60-0.86) | 0.79 (0.61-0.91) | |
| F M1 S1 | -5352 | -1231 | -1013 | -778 | -602 | 0.70 (0.52-0.81) | 0.87 (0.77-0.94) | 0.80 (0.61-0.88) | 0.80 (0.71-0.86) | 0.79 (0.67-0.86) | 0.80 (0.68-0.87) | |
| F T1 M1 | -5443 | -1211 | -1003 | -751 | -577 | 0.71 (0.53-0.81) | 0.88 (0.75-0.93) | 0.80 (0.71-0.87) | 0.81 (0.72-0.88) | 0.79 (0.69-0.85) | 0.79 (0.62-0.88) | |
| F P1 M1 | -5416 | -1151 | -992 | -741 | -492 | 0.70 (0.54-0.81) | 0.88 (0.76-0.93) | 0.79 (0.57-0.88) | 0.79 (0.62-0.87) | 0.79 (0.69-0.86) | 0.80 (0.66-0.88) | |
| S M1 E1 | -5179 | -1390 | -1151 | -940 | -770 | 0.69 (0.53-0.81) | 0.87 (0.76-0.94) | 0.87 (0.75-0.93) | 0.87 (0.75-0.94) | 0.87 (0.75-0.94) | 0.56 (0.49-0.61) | |
| S M1 F1 | -2655 | -1203 | -997 | -756 | -562 | 0.52 (0.25-0.77) | 0.90 (0.81-0.94) | 0.88 (0.77-0.94) | 0.87 (0.75-0.93) | 0.88 (0.76-0.93) | 0.76 (0.57-0.89) | |
| S M1 S1 | -2785 | -1328 | -1133 | -887 | -675 | 0.53 (0.26-0.77) | 0.90 (0.82-0.95) | 0.88 (0.78-0.93) | 0.87 (0.77-0.91) | 0.87 (0.76-0.93) | 0.76 (0.59-0.90) | |
| S T1 M1 | -2683 | -1304 | -1105 | -912 | -674 | 0.53 (0.25-0.78) | 0.90 (0.81-0.96) | 0.87 (0.76-0.93) | 0.88 (0.77-0.94) | 0.88 (0.76-0.94) | 0.77 (0.60-0.89) | |
| S P1 M1 | -2709 | -1260 | -1052 | -860 | -662 | 0.53 (0.26-0.77) | 0.90 (0.81-0.95) | 0.88 (0.77-0.95) | 0.87 (0.75-0.93) | 0.89 (0.77-0.94) | 0.76 (0.59-0.89) | |
| T M1 E1 | 215 | -1256 | -1042 | -817 | -633 | 0.22 (0.06-0.40) | 0.91 (0.80-0.95) | 0.89 (0.79-0.93) | 0.90 (0.80-0.94) | 0.89 (0.79-0.93) | 0.87 (0.74-0.94) | |
| T M1 F1 | 1097 | -1120 | -952 | -717 | -513 | 0.09 (0.00-0.30) | 0.91 (0.79-0.97) | 0.91 (0.79-0.95) | 0.91 (0.79-0.95) | 0.86 (0.75-0.91) | 0.93 (0.83-0.98) | |
| T M1 S1 | 1052 | -1256 | -1050 | -856 | -628 | 0.09 (0.03-0.30) | 0.91 (0.79-0.96) | 0.89 (0.78-0.94) | 0.89 (0.78-0.94) | 0.85 (0.74-0.90) | 0.93 (0.83-0.98) | |
| T T1 M1 | 948 | -1227 | -1042 | -827 | -632 | 0.11 (0.05-0.30) | 0.92 (0.80-0.97) | 0.90 (0.79-0.95) | 0.88 (0.77-0.93) | 0.83 (0.74-0.88) | 0.93 (0.84-0.97) | |
| T P1 M1 | 1027 | -1169 | -1006 | -753 | -581 | 0.10 (0.03-0.30) | 0.91 (0.81-0.96) | 0.89 (0.78-0.95) | 0.90 (0.79-0.95) | 0.84 (0.69-0.89) | 0.93 (0.83-0.98) | |
| P M1 E1 | -3280 | -1215 | -983 | -759 | -596 | 0.57 (0.45-0.68) | 0.89 (0.77-0.95) | 0.86 (0.75-0.90) | 0.86 (0.75-0.90) | 0.86 (0.75-0.90) | 0.76 (0.57-0.86) | |
| P M1 F1 | -3129 | -1085 | -885 | -710 | -479 | 0.56 (0.44-0.64) | 0.87 (0.72-0.94) | 0.85 (0.72-0.90) | 0.86 (0.73-0.92) | 0.84 (0.72-0.89) | 0.75 (0.56-0.85) | |
| P M1 S1 | -3127 | -1230 | -1028 | -802 | -562 | 0.56 (0.46-0.64) | 0.87 (0.72-0.95) | 0.84 (0.71-0.89) | 0.85 (0.72-0.90) | 0.85 (0.72-0.89) | 0.74 (0.54-0.85) | |
| P T1 M1 | -3166 | -1186 | -985 | -710 | -538 | 0.56 (0.46-0.65) | 0.88 (0.74-0.95) | 0.85 (0.72-0.91) | 0.81 (0.68-0.87) | 0.84 (0.72-0.89) | 0.76 (0.58-0.86) | |
| P P1 M1 | -3149 | -1143 | -950 | -767 | -557 | 0.56 (0.46-0.64) | 0.88 (0.74-0.94) | 0.86 (0.74-0.91) | 0.86 (0.73-0.91) | 0.82 (0.72-0.87) | 0.77 (0.59-0.86) | |
| F1 E1 E | -4642 | -1312 | - | - | - | 0.66 (0.53-0.77) | 0.91 (0.79-0.96) | - | - | - | 0.63 (0.55-0.70) | |

^a^ BIC: Bayesian Information Criterion; ^b^ Nash-Sutcliffe coefficient of efficiency index, values presented are calculated for the whole area (values in parenthesis represent range for polygons); ^c^ Canonical correlation coefficients for the first common trend of Models I and II, values presented are calculated for the whole area (values in parenthesis represent range for polygons).
