# Supplementary material for: Beyond Precipitation: Physiographic Gradients Dictate the Relative Importance of Environmental Drivers on Savanna Vegetation
Source: PLoS One. 2013 Aug 30;8(8):e72348. doi: 10.1371/journal.pone.0072348 (PMC3758306; doi:10.1371/journal.pone.0072348)
Supplement: Table S3 — Spatial distribution of weighting coefficients for model III. (DOCX) [file pone.0072348.s003.docx]

**Table S3.** Spatial distribution of weighting coefficients for model III. Bold values are significant for t-values > 2.

|  |  | FSPTE | | | | | | |
| --- | --- | --- | --- | --- | --- | --- | --- | --- |
| Watershed | MAP (mm) | μ | β_F_ | β_S_ | β_P_ | β_T_ | β_E_ |  |
| Okavango | < 450 | 0.00 | **-0.13** | **0.59** | **0.42** | -0.01 | -0.01 |  |
|  | 450-500 | 0.00 | **-0.16** | **0.64** | **0.26** | 0.04 | -0.06 |  |
|  | 500-550 | 0.00 | **-0.21** | **0.59** | **0.26** | 0.08 | -0.08 |  |
|  | 550-600 | 0.00 | **-0.20** | **0.51** | **0.18** | **0.15** | **-0.22** |  |
|  | 600-650 | 0.00 | **-0.22** | **0.43** | **0.20** | **0.17** | **-0.28** |  |
|  | 650-700 | 0.00 | **-0.25** | **0.38** | **0.19** | **0.19** | **-0.29** |  |
|  | 700-750 | 0.00 | **-0.27** | **0.38** | **0.18** | **0.19** | **-0.28** |  |
|  | 750-800 | 0.00 | **-0.30** | **0.31** | **0.18** | **0.22** | **-0.33** |  |
|  | 800-850 | 0.00 | **-0.38** | **0.30** | **0.19** | **0.19** | **-0.24** |  |
|  | 850-900 | 0.00 | **-0.42** | **0.35** | **0.18** | **0.15** | **-0.16** |  |
|  | 900-950 | 0.00 | **-0.48** | **0.22** | **0.10** | **0.27** | **-0.28** |  |
|  | 950-1000 | 0.00 | **-0.47** | **0.14** | **0.13** | **0.38** | **-0.26** |  |
|  | 1000-1050 | 0.00 | **-0.52** | **0.16** | **0.12** | **0.32** | **-0.24** |  |
|  | 1050-1100 | 0.00 | **-0.44** | **0.14** | **0.12** | **0.45** | **-0.25** |  |
|  | 1100-1150 | 0.00 | **-0.44** | **0.13** | **0.14** | **0.47** | **-0.22** |  |
|  | 1150-1200 | 0.00 | **-0.40** | **0.16** | **0.12** | **0.48** | **-0.26** |  |
|  | 1200-1250 | 0.00 | **-0.42** | **0.16** | **0.13** | **0.43** | **-0.28** |  |
|  | 1250-1300 | 0.00 | **-0.61** | 0.01 | **0.11** | **0.32** | **-0.23** |  |
| Kwando | < 450 | 0.00 | **-0.14** | **0.54** | **0.32** | 0.00 | -0.13 |  |
|  | 450-500 | 0.00 | **-0.17** | **0.59** | **0.32** | -0.02 | -0.06 |  |
|  | 500-550 | 0.00 | **-0.25** | **0.49** | **0.28** | 0.07 | **-0.13** |  |
|  | 550-600 | 0.00 | **-0.23** | **0.49** | **0.30** | 0.08 | -0.13 |  |
|  | 600-650 | 0.00 | **-0.23** | **0.48** | **0.28** | **0.11** | **-0.16** |  |
|  | 650-700 | 0.00 | **-0.25** | **0.44** | **0.25** | **0.19** | **-0.20** |  |
|  | 700-750 | 0.00 | **-0.25** | **0.45** | **0.22** | **0.20** | **-0.21** |  |
|  | 750-800 | 0.00 | **-0.31** | **0.38** | **0.19** | **0.19** | **-0.25** |  |
|  | 800-850 | 0.00 | **-0.38** | **0.31** | **0.14** | **0.19** | **-0.27** |  |
|  | 850-900 | 0.00 | **-0.42** | **0.24** | **0.14** | **0.20** | **-0.30** |  |
|  | 900-950 | 0.00 | **-0.45** | **0.18** | **0.14** | **0.22** | **-0.31** |  |
|  | 950-1000 | 0.00 | **-0.50** | 0.09 | **0.14** | **0.24** | **-0.33** |  |
|  | 1000-1050 | 0.00 | **-0.53** | 0.06 | **0.11** | **0.31** | **-0.31** |  |
|  | 1050-1100 | 0.00 | **-0.52** | 0.06 | **0.12** | **0.35** | **-0.27** |  |
| Zambezi | 550-600 | 0.00 | **-0.16** | **0.54** | **0.41** | 0.00 | -0.04 |  |
|  | 600-650 | 0.00 | **-0.25** | **0.35** | **0.34** | **0.10** | **-0.19** |  |
|  | 650-700 | 0.00 | **-0.28** | **0.26** | **0.28** | **0.12** | **-0.31** |  |
|  | 700-750 | 0.00 | **-0.29** | **0.26** | **0.26** | **0.15** | **-0.30** |  |
|  | 750-800 | 0.00 | **-0.32** | **0.27** | **0.23** | **0.18** | **-0.31** |  |
|  | 800-850 | 0.00 | **-0.35** | **0.30** | **0.19** | **0.19** | **-0.27** |  |
|  | 850-900 | 0.00 | **-0.30** | **0.26** | **0.19** | **0.18** | **-0.37** |  |
|  | 900-950 | 0.00 | **-0.31** | **0.25** | **0.15** | **0.19** | **-0.40** |  |
|  | 950-1000 | 0.00 | **-0.39** | **0.18** | **0.12** | **0.20** | **-0.40** |  |
|  | 1000-1050 | 0.00 | **-0.40** | **0.18** | **0.09** | **0.21** | **-0.42** |  |
|  | 1050-1100 | 0.00 | **-0.43** | **0.15** | **0.09** | **0.27** | **-0.40** |  |
|  | 1100-1150 | 0.00 | **-0.49** | 0.04 | **0.10** | **0.32** | **-0.38** |  |
|  | 1150-1200 | 0.00 | **-0.49** | 0.06 | **0.11** | **0.30** | **-0.37** |  |
|  | 1200-1250 | 0.00 | **-0.47** | **0.09** | **0.11** | **0.31** | **-0.36** |  |
|  | 1250-1300 | 0.00 | **-0.48** | 0.08 | **0.12** | **0.31** | **-0.35** |  |
|  | > 1300 | 0.00 | **-0.46** | 0.03 | **0.12** | **0.41** | **-0.34** |  |
