# Supplementary material for: Beyond Precipitation: Physiographic Gradients Dictate the Relative Importance of Environmental Drivers on Savanna Vegetation
Source: PLoS One. 2013 Aug 30;8(8):e72348. doi: 10.1371/journal.pone.0072348 (PMC3758306; doi:10.1371/journal.pone.0072348)
Supplement: Appendix S1 — Collinearity test of explanatory variables. (DOCX) [file pone.0072348.s004.docx]

**Appendix S1**

**Collinearity test of explanatory variables**

Nine explanatory variables were initially explored consisting of monthly times series of precipitation, mean temperature, minimum temperature, maximum temperature, soil moisture, fire, relative humidity, actual evapotranspiration and potential evapotranspiration (Table A1).

**Table A1.** Initial set of candidate explanatory variables and their sources.

| Data set | Symbol | | Source |
| --- | --- | --- | --- |
| Matsuura and Willmott’ s Monthly Precipitation | | P | http://climate.geog.udel.edu/~climate/html_pages/download.html#P2011 |
| Matsuura and Willmott’ s Monthly Temperature | | T | http://climate.geog.udel.edu/~climate/html_pages/download.html#P2011 |
| NCEP-DOE Reanalysis II Monthly Maximum Temperature | | M | http://www.esrl.noaa.gov/psd/data/gridded/data.ncep.reanalysis2.gaussian.html |
| NCEP-DOE Reanalysis II Monthly Minimum Temperature | | m | http://www.esrl.noaa.gov/psd/data/gridded/data.ncep.reanalysis2.gaussian.html |
| NCEP-DOE Reanalysis II Monthly  Relative Humidity | | RH | http://www.esrl.noaa.gov/psd/data/gridded/data.ncep.reanalysis2.pressure.html |
| Actual Evapotranspiration | | Ea | <http://www.ntsg.umt.edu/project/et> |
| NCEP-DOE Reanalysis II Monthly Potential Evapotranspiration | | E | http://www.esrl.noaa.gov/psd/data/gridded/data.ncep.reanalysis2.gaussian.html |
| CPC Monthly Soil Moisture | | S | http://www.esrl.noaa.gov/psd/data/gridded/tables/monthly.html |
| Monthly MODIS Thermal Anomalies & Fire ([MOD14A2](https://lpdaac.usgs.gov/products/modis_products_table/mod14a2)) | | F | <http://reverb.echo.nasa.gov> |

Following Zuur *et al.* (2007) the variance inflation factor (VIF) >10 is an indicator of high collinearity among explanatory variables, i.e. the variation in one variable is well explained by the other variables. The authors recommend using a backward selection method to remove one variable at a time (the one with the highest VIF >10) and recalculate VIF after each iteration (Step 1-4 in Table A2) until we obtain a set of non-collinear variables, i.e. all with VIF<10 (Step 4 in Table A2).

Finally, while the use of Ea might be preferable in the analysis, Mu *et al.* (2011) show that NDVI values are used in the calculation of MODIS-based Ea (eqs. 9-12 in Mu *et al.* paper). This functional dependence makes it less desirable in the analysis that the more independent remote sensing estimate of E. However, notice that both Ea and E are considered in the initial set of variables but the collinearity test (VIF>10) eliminates Ea in favor of E in the ensuing DFA.

**Table A2.** Test of collinearity of explanatory variables. The final set of explanatory variables selected based on VIF<10 are shown in bold. VIF values are calculated for area-weighted values for the region.

| Group | Symbol^1^ | VIF | | | |
| --- | --- | --- | --- | --- | --- |
|  |  | Step 1 | Step 2 | Step 3 | Step 4 |
| Precipitation | **P** | 6.6 | 5.1 | 3.86 | **2.8** |
| Temperature | **T** | 13.9 | 12.5 | 8.69 | **2.7** |
|  | m | 15.0 | 11.5 | 11.4 | **-** |
|  | **M** | 12.0 | 4.0 | 3.9 | **3.5** |
| Relative Humidity | RH | 47.0 | - | - | **-** |
| Evapotranspiration | Ea | 20.8 | 14.8 | - | **-** |
|  | **E** | 13.3 | 10.3 | 7.8 | **6.9** |
| Soil Moisture | **S** | 4.9 | 4.7 | 3.4 | **3.4** |
| Fire | **F** | 3.8 | 3.0 | 3.0 | **2.7** |

^1^ Explanatory variables are: precipitation (P), mean temperature (T), minimum temperature (m), maximum temperature (M), actual evapotranspiration (Ea), potential evapotranspiration (E), relative humidity (RH), soil moisture (S) and fire (F).

**References:**

Mu Q, Zhao M, Running SW (2011) Improvements to a MODIS global terrestrial evapotranspiration algorithm. Remote Sens Environ 115:1781–1800.

Zuur AF, Ieno EN, Smith GM (2007) Analyzing ecological data. New York: Springer. 672 p.
