# Supplementary material for: Beyond Precipitation: Physiographic Gradients Dictate the Relative Importance of Environmental Drivers on Savanna Vegetation
Source: PLoS One. 2013 Aug 30;8(8):e72348. doi: 10.1371/journal.pone.0072348 (PMC3758306; doi:10.1371/journal.pone.0072348)
Supplement: Appendix S2 — Herbivory in the study area. (DOCX) [file pone.0072348.s005.docx]

**Appendix S2**

**Herbivory in the study region**

The effect of herbivory in the Savanna landscape will vary depending on the specific study region and spatial and temporal scale of the analysis. Based on FAO gridded data (Wint and Robinson, 2007) the cattle stocking rate in our study region is very low (Fig. R2-A), with typical values at the pixel level between <1-10 head/km^2^ (Fig. S1A), and a range of <1-6 head/km^2^ at the polygon level used in the analysis (Fig. S1B) compared to the highest stocking rates of >250 head/km^2^ in other African savanna regions outside the study area. In particular, although a large portion of Botswana’s total land area is used for communal grazing, the largest cattle concentration is away from the study area in southeastern Botswana, with very low stocking in the Okavango Delta area (Fig. S1A). In terms of wildlife herbivory, the largest herbivore density nearby is outside the study region (Chobe National Park, Botswana), and although there are a number of wildlife management areas throughout the region, these are of relatively small size, suggesting their effect is likely minimal at the polygon level.

| 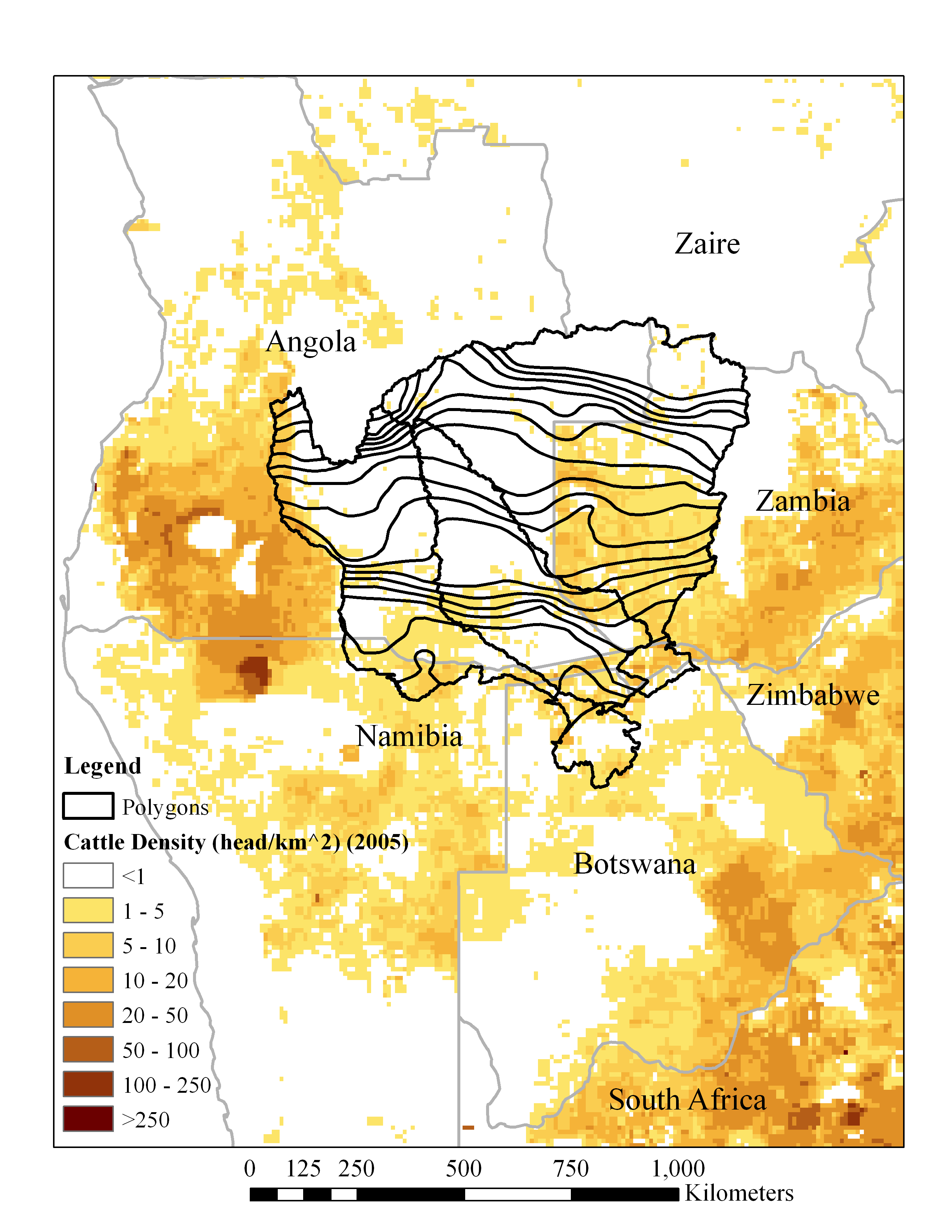  (A) |   (B) |
| --- | --- |
|  |  |

Figure A1. Gridded cattle density in study region (Wint and Robinson, 2007) at (A) the pixel scale; and (B) aggregated at the polygon scale used in the analysis.

In general, herbivores remove vegetation as a function of vegetation biomass and herbivore density (e.g., Beisner, 2012), with the availability of high-quality forage material as the major constraint on herbivores’ biomass consumption rates (e.g., Zweifel-Schielly *et al.*, 2012). Thus, at the low densities present in the study region, herbivory likely reduces proportionally the amount of green vegetation in small quantities, following primarily the same seasonal cycling as plant green-up and senescence. The result is that while herbivory might act to slightly reduce the magnitude of NDVI it does likely not change the temporal pattern (i.e., the variance) observed at the monthly time scale pursued in this study. Barring large-scale introduction, exclusion, or migration of large populations of herbivores (for which we could find no evidence during the study period), our approach to deconstructing the variance in the normalized, spatially distributed NDVI time series focuses on those variables that have clear temporal variation.

Finally, we note that significant herbivory differences across the region (if present) would be captured implicitly as latent effects contained in the common trends (unexplained variance) in Model II, with spatial differences across the landscape represented by the loading factors for each polygon. That the removal of the common trends in Model III did not negatively affect model predictions supports the relatively low importance of latent effects (and herbivory) in the analysis for this region.

**References:**

Beisner BE (2012) Alternative Stable States. Nature Education Knowledge 3: 33.

Wint GRW and Robinson TP (2007) Gridded livestock of the world 2007. Rome: FAO. 131 p. Available: <http://harvestchoice.org/about/harvestchoice/intellect.html>. Accessed 1 June 2013.

Zweifel‐Schielly B, Leuenberger Y, Kreuzer M, Suter W (2012) A herbivore's food landscape: seasonal dynamics and nutritional implications of diet selection by a red deer population in contrasting Alpine habitats. J Zool 286: 68-80. doi: 10.1111/j.1469-7998.2011.00853.x.
